# Synthesis and Preclinical Evaluation of Two Novel ^68^Ga-Labeled Bispecific PSMA/FAP-Targeted Tracers with 2-Nal-Containing PSMA-Targeted Pharmacophore and Pyridine-Based FAP-Targeted Pharmacophore

**DOI:** 10.3390/molecules29040800

**Published:** 2024-02-09

**Authors:** Arsyangela Verena, Helen Merkens, Chao-Cheng Chen, Devon E. Chapple, Lei Wang, Shreya Bendre, Antonio A. W. L. Wong, François Bénard, Kuo-Shyan Lin

**Affiliations:** 1 Department of Molecular Oncology, BC Cancer Research Institute, Vancouver, BC V5Z1L3, Canada; arsya@mail.ubc.ca (A.V.); hmerkens@bccrc.ca (H.M.); ccchen@bccrc.ca (C.-C.C.); dchapple@bccrc.ca (D.E.C.); lewang@bccrc.ca (L.W.); sbendre@bccrc.ca (S.B.); antwong@bccrc.ca (A.A.W.L.W.); fbenard@bccrc.ca (F.B.); 2 Department of Molecular Imaging and Therapy, BC Cancer, Vancouver, BC V5Z4E6, Canada; 3 Department of Radiology, University of British Columbia, Vancouver, BC V5Z1M9, Canada

**Keywords:** bispecific radiotracers, PSMA, FAP, PET/CT, gallium-68, prostate cancer

## Abstract

Some bispecific radiotracers have been developed to overcome the limitations of monospecific tracers and improve detection sensitivity for heterogeneous tumor lesions. Here, we aim to synthesize two bispecific tracers targeting prostate-specific membrane antigen (PSMA) and fibroblast activation protein (FAP), which are key markers expressed in prostate cancer. A pyridine-based FAP-targeted ligand was synthesized through multi-step organic synthesis and then connected to the 2-Nal-containing PSMA-targeted motif. The K_i_(PSMA) values of Ga-complexed bispecific ligands, Ga-AV01084 and Ga-AV01088, were 11.6 ± 3.25 and 28.7 ± 6.05 nM, respectively, and the IC_50_(FAP) values of Ga-AV01084 and Ga-AV01088 were 10.9 ± 0.67 and 16.7 ± 1.53 nM, respectively. Both [^68^Ga]Ga-AV01084 and [^68^Ga]Ga-AV01088 enabled the visualization of PSMA-expressing LNCaP tumor xenografts and FAP-expressing HEK293T:hFAP tumor xenografts in PET images acquired at 1 h post-injection. However, the tumor uptake values from the bispecific tracers were still lower than those obtained from the monospecific tracers, PSMA-targeted [^68^Ga]Ga-PSMA-617 and FAP-targeted [^68^Ga]Ga-AV02070. Further investigations are needed to optimize the selection of linkers and targeted pharmacophores to improve the tumor uptake of bispecific PSMA/FAP tracers for prostate cancer imaging.

## 1. Introduction

As the second most common cancer and the fifth leading cause of cancer death in men worldwide, prostate cancer had an estimated 1,410,000 new cases and 375,304 deaths in 2020 [1]. Based on the recent cancer statistics by Siegel et al. [2], the 5-year relative survival rates of localized and regional prostate cancer are >99%; however, they have decreased significantly to 32% for metastatic prostate cancer patients. Therefore, early and accurate detection is important to further improve the survival rates of prostate cancer patients. Recently, the application of radiopharmaceuticals for the non-invasive detection and treatment of prostate cancer has shown positive outcomes, such as improving positive lesion detection rate and prolonging imaging-based progression-free survival [3,4]. One of the promising radiotracers for metastatic prostate cancer diagnosis, which has been approved by the US FDA, is [^68^Ga]Ga-PSMA-11, which targets prostate-specific membrane antigen (PSMA) [5,6]. PSMA is a type II transmembrane glycoprotein, also known as folate hydrolase 1 and glutamate carboxypeptidase II [7,8]. PSMA has been found to be highly expressed in prostate tumors and the neovasculature of other types of cancer, such as oral squamous cell, breast, and renal cancers [9,10,11]. Therefore, many attempts to develop PSMA-targeted radioligands for prostate cancer imaging and radioligand therapy have been made.

Clinical data have shown that the PSMA-targeted radioligand therapeutic agent, [^177^Lu]Lu-PSMA-617, has good efficacy in treating metastatic castration-resistant prostate cancer (mCRPC) patients [3]. However, patients with low to no PSMA-expressing lesions are ineligible to benefit from this treatment [12,13]. This intra-patient heterogeneous PSMA expression in prostate cancer lesions can lead to worse overall survival due to PSMA-negative lesions being misdiagnosed as false-negative [14,15]. One strategy to improve lesion detection in this patient cohort is using bispecific radioligands to target PSMA and other overexpressed proteins in prostate cancer lesions, such as fibroblast activation protein (FAP). FAP is a type II transmembrane serine protease [16] that is overexpressed by cancer-associated fibroblasts (CAFs) in the tumor microenvironment (TME) [17]. It is found to be highly expressed in 90% of epithelial tumors, including prostate cancer [18,19], and its expression is associated with worse prognosis [20], making it a promising target for cancer diagnosis and therapy. In fact, there are many reports on the development of new promising FAP-targeted radioligands and some novel radioligands are being validated clinically, such as [^68^Ga]Ga-FAPI-04 [21], [^68^Ga]Ga-FAPI-46 [22], and [^177^Lu]Lu-FAP-2286 [23]. Despite the potential of FAP-targeted radioligands shown in these studies, their tumor retention and efficacy have not been promising in the clinic. Hence, by targeting PSMA, which is overexpressed in cancer cells, and FAP, which is overexpressed in tumor stroma, we hypothesized that it would potentially increase the lesion detection rate and tumor retention in prostate cancer patients.

Previous studies have demonstrated the potential of utilizing bispecific radioligands to increase the detection sensitivity of prostate cancer imaging, such as bispecific PSMA/gastrin-releasing peptide receptor (PSMA/GRPR) radioligands that have higher tumor uptake in PSMA- and GRPR-expressing mouse tumor models compared to monospecific radiotracers [24]. Recently, several bispecific PSMA/FAP radioligands have been reported: ^64^Cu-, ^18^F-, and ^68^Ga-labeled bispecific PSMA/FAP radiotracers by Boinapally et al. [25], Hu et al. [26], and Wang et al. [27], respectively. Their studies confirmed the potential usage of bispecific PSMA/FAP ligands to improve tumor uptake in PSMA- and FAP-expressing tumor models in comparison to the monospecific counterparts.

Recently, our group also developed three ^68^Ga-labeled bispecific PSMA/FAP radiotracers, which incorporate a quinoline-based FAP pharmacophore and an anthracene-containing PSMA pharmacophore [28]. We observed a high blood uptake (5–12 %ID/g at 1 h post-injection) in a mouse model with decreased tumor uptake in comparison to the ^68^Ga-labeled monospecific counterparts, PSMA-targeted [^68^Ga]Ga-HTK03041 and FAP-targeted [^68^Ga]Ga-FAPI-04. We suspected that the longer blood retention could be caused by the increased lipophilicity of the ligands and potential interactions, such as π–π stacking between the quinoline ring and anthracene ring, which hindered the binding of tracers to FAP and PSMA, leading to decreased tumor uptake. Our goal in this study is to solve these issues and improve the binding affinity and tumor uptake by replacing the pharmacophores with less lipophilic motifs, such as changing part of the PSMA-targeted pharmacophore from Ala(9-Anth) to 2-Nal and changing the quinoline motif in the FAP-targeted pharmacophore to a more hydrophilic pyridine.

**Figure 1:**
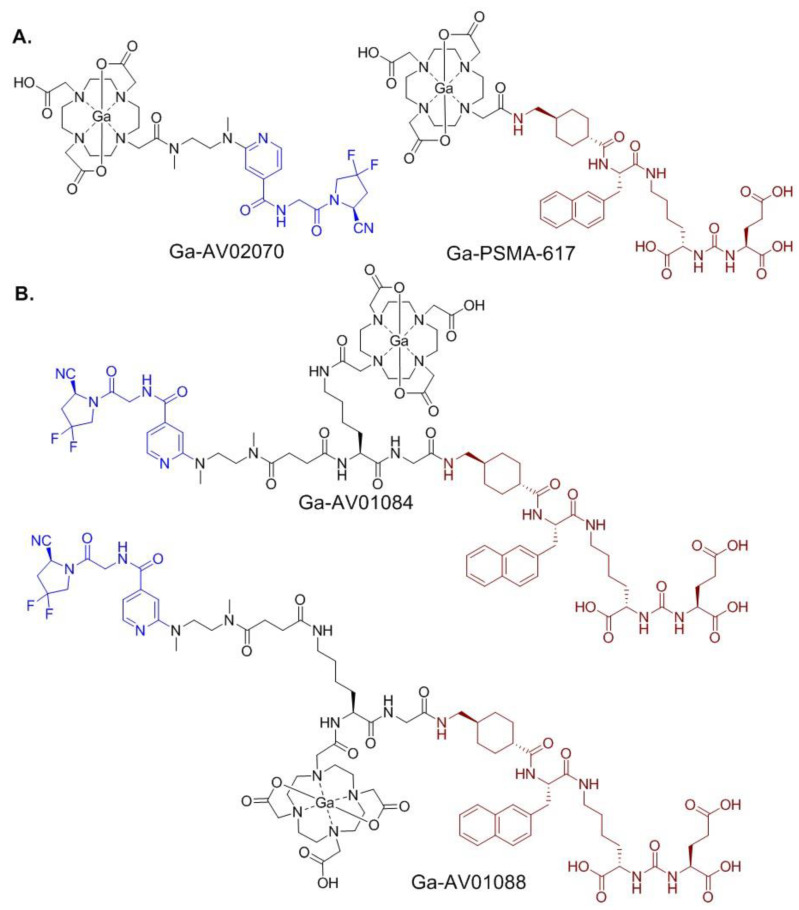
Chemical structures of (**A**) PSMA-targeted [^68^Ga]Ga-PSMA-617 and FAP-targeted [^68^Ga]Ga-AV02070; and (**B**) bispecific PSMA/FAP-targeted [^68^Ga]Ga-AV01084 and [^68^Ga]Ga-AV01088. The PSMA- and FAP-targeted pharmacophores are shown in brown and blue, respectively.

Many potent pyridine-based FAP inhibitors (FAPIs) have been reported [29,30]; however, there are still few reports on the development of pyridine-based FAP-targeted tracers. Previously, we synthesized and evaluated two novel ^68^Ga-labeled pyridine-based FAP-targeted tracers, [^68^Ga]Ga-AV02053 and [^68^Ga]Ga-AV02070, and compared their binding affinity and tumor uptake to those of [^68^Ga]Ga-FAPI-04 [31]. We discovered that although [^68^Ga]Ga-FAPI-04 has a higher binding affinity toward FAP and higher tumor uptake, both of our pyridine-based tracers have lower uptake in blood, muscle, and bone, leading to much higher tumor-to-background contrast ratios. Our results suggest that pyridine-based FAPIs are more hydrophilic than quinoline-based FAPIs and have potential to help reduce blood uptake. Furthermore, we found that Ga-AV02070 (Figure 1A), which has a carbonyl group at the para position to the pyridine nitrogen, has a better binding affinity to FAP and a higher tumor uptake compared to Ga-AV02053, which has a carbonyl group at the meta position to the pyridine nitrogen. Hence, the pharmacophore of AV02070 is a potential candidate for the design of FAP-targeted tracers.

In this paper, we report the design, synthesis, and evaluation of two bispecific PSMA/FAP-targeted radiotracers, [^68^Ga]Ga-AV01084 and [^68^Ga]Ga-AV01088 (Figure 1B). The PSMA-binding motif of AV01084 and AV01088 was based on the 2-Nal-containing PSMA-targeted tracer, [^68^Ga]Ga-PSMA-617 (Figure 1A) [32], and their FAP-targeted motif was derived from our pyridine-based FAP-targeted radiotracer, [^68^Ga]Ga-AV02070 (Figure 1A). The difference between the two tracers is the position of the DOTA chelator linked to the lysine, which is the ε-amino group in AV01084 and the α-amino group in AV01088. We performed an in vitro competition binding assay, PET imaging, and ex vivo biodistribution studies in preclinical PSMA-expressing LNCaP and FAP-expressing HEK293T:hFAP tumor models to evaluate the potential of [^68^Ga]Ga-AV01084 and [^68^Ga]Ga-AV01088 for prostate cancer imaging. The results were then compared with those of the corresponding monospecific tracers, [^68^Ga]Ga-PSMA-617 and [^68^Ga]Ga-AV02070.

## 2. Results

### 2.1. Synthesis of Bispecific PSMA/FAP Ligand

**Scheme 1:**
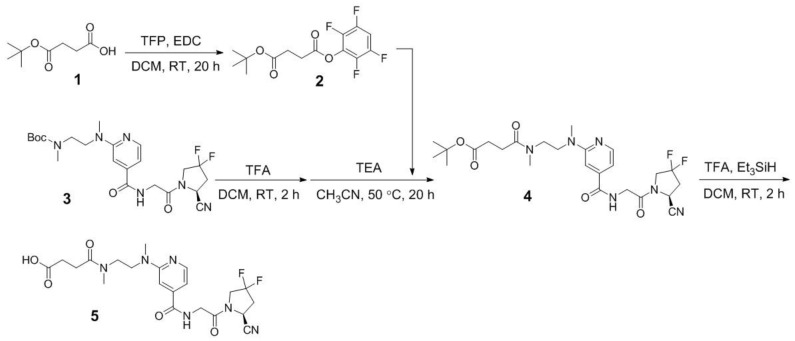
Synthesis of (*S*)-4-((2-((4-((2-(2-cyano-4,4-difluoropyrrolidin-1-yl)-2-oxoethyl) carbamoyl)pyridin-2-yl)(methyl)amino)ethyl)(methyl)amino)-4-oxobutanoic acid (**5**).

AV01084 and AV01088 (Figure 1B) were synthesized on solid phase. Briefly, Lys(Lys(ivDde)-Gly-tranexamic acid-2-Nal)-urea-Glu(OtBu)-OtBu was first synthesized on solid phase, followed by amide coupling of the FAP-targeted motif, compound **5**, and the DOTA chelator. To synthesize compound **5** (Scheme 1), compound **1** was first coupled with 2,3,5,6-tetrafluorophenol (TFP) to obtain the activated ester **2** in 71% yield. Compound **3,** which was synthesized following literature procedures [31], was first Boc-deprotected using trifluoroacetic acid (TFA), followed by coupling with compound **2** to obtain compound **4** in a 45% yield. The *t*-Butyl-protecting group of compound **4** was removed using TFA and compound **5** was obtained as a TFA salt.

To synthesize AV01084, compound **5** was coupled to Lys(Lys(ivDde)-Gly-tranexamic acid-2-Nal)-urea-Glu(OtBu)-OtBu, followed by removal of the ivDde group at the Lys side chain and subsequent coupling with the DOTA chelator. To synthesize AV01088, DOTA chelator was first coupled to Lys(Lys(ivDde)-Gly-tranexamic acid-2-Nal)-urea-Glu(OtBu)-OtBu, followed by the deprotection of the amino group at the Lys side chain and coupling with compound **5**. The DOTA-conjugated ligands were then cleaved off from resin and purified by HPLC (Table S1). AV01084 and AV01088 were obtained in 12% and 7.2% yields, respectively.

Detailed syntheses and characterizations of ^nat^Ga- and ^68^Ga-labeled analogs of AV01084 and AV01088 are described in the Supplementary Materials (also see Tables S2 and S3). ^nat^Ga-labeled AV01084 and AV01088 were obtained in 60% and 21% yields, respectively. ^68^Ga-labeled AV01084 and AV01088 were obtained in 33–64% decay-corrected radiochemical yields with >95% radiochemical purity and ≥44 GBq/µmol molar activity.

### 2.2. Binding Affinity and Lipophilicity

**Figure 2:**
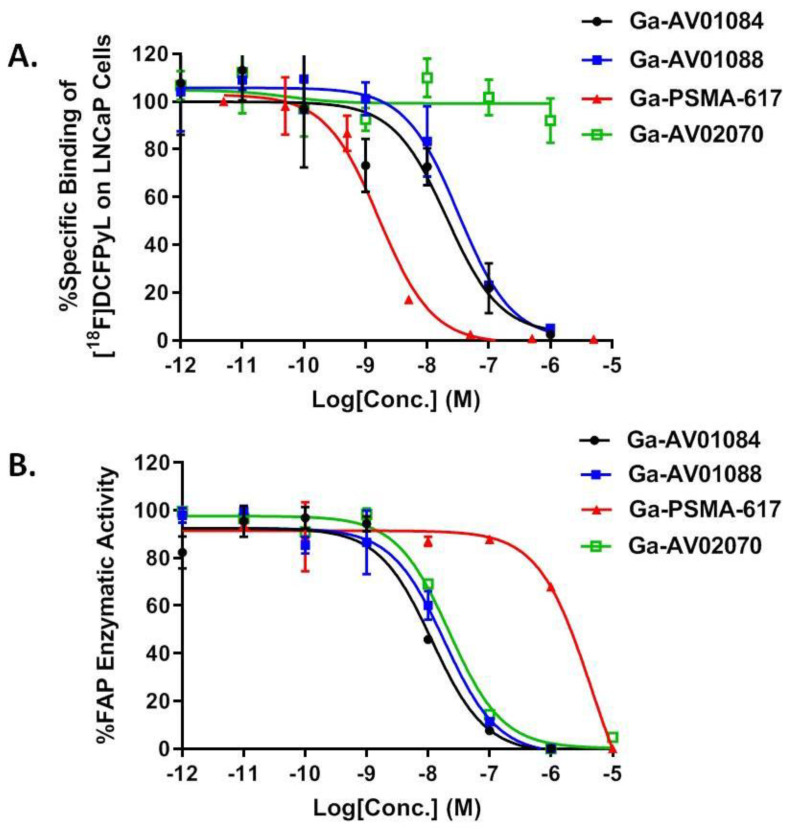
(**A**) Displacement curves of [^18^F]DCFPyL by Ga-AV01084, Ga-AV01088, Ga-AV02070, and Ga-PSMA-617 generated using PSMA-expressing LNCaP cells; (**B**) Fluorescence curves of FAP enzymatic activity on Suc-Gly-Pro-AMC substrate with inhibition by Ga-AV01084, Ga-AV01088, Ga-AV02070, and Ga-PSMA-617.

The binding affinities of Ga-AV01084, Ga-AV01088, and Ga-AV02070 to PSMA were measured by a cell-based competition binding assay using PSMA-expressing LNCaP prostate cancer cells and were compared to that of the previously published Ga-PSMA-617 (K_i_ = 1.23 ± 0.08 nM) [33]. These ligands inhibited the binding of [^18^F]DCFPyL to LNCaP cells in a dose-dependent manner (Figure 2A) and the calculated K_i_(PSMA) values for Ga-AV01084, Ga-AV01088, and Ga-AV02070 were 11.6 ± 3.25, 28.7 ± 6.05, and >1000 nM, respectively (*n* = 3).

Enzyme inhibition assays were performed using Suc-Gly-Pro-AMC as the FAP substrate to determine the binding affinities of Ga-AV01084, Ga-AV01088, and Ga-PSMA-617 to human FAP, and their data were compared to that of the previously published Ga-AV02070 (IC_50_ = 17.1 ± 4.6 nM) [31]. The human FAP enzymatic activity on the substrate was inhibited by these ligands in a dose-dependent manner (Figure 2B). The calculated IC_50_ values for Ga-AV01084, Ga-AV01088, and Ga-PSMA-617 were 10.9 ± 0.67, 16.7 ± 1.53 and >1000 nM, respectively (*n* = 3).

The lipophilicity of the ^68^Ga-labeled AV01084 and AV01088 were calculated using LogD_7.4_ measurement. The LogD_7.4_ values of [^68^Ga]Ga-AV01084 and [^68^Ga]Ga-AV01088 were −3.61 ± 0.07 and −3.66 ± 0.25, respectively. The values indicate that both tracers are highly hydrophilic.

### 2.3. PET Imaging, Ex Vivo Biodistribution, and Blocking Studies

**Figure 3:**
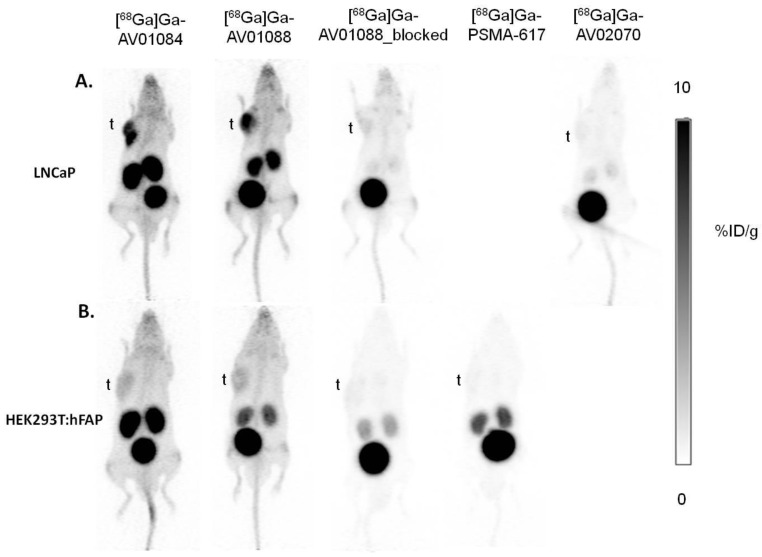
Representative maximum-intensity-projection PET images of [^68^Ga]Ga-AV01084, [^68^Ga]Ga-AV01088, [^68^Ga]Ga-PSMA-617, and [^68^Ga]Ga-AV02070 acquired at 1 h post-injection in mice bearing (**A**) LNCaP tumor xenografts and (**B**) HEK293T:hFAP tumor xenografts. The blocking with 2-PMPA (in LNCaP tumor xenografts) and FAPI-04 (in HEK293T:hFAP tumor xenografts) decreased the tumor uptake to the background level. t: tumor.

Representative PET images acquired at 1 h post-injection using [^68^Ga]Ga-AV01084, [^68^Ga]Ga-AV01088, [^68^Ga]Ga-PSMA-617, and [^68^Ga]Ga-AV02070 are provided in Figure 3. High uptake of all tracers in the kidneys and bladder indicated that they were excreted primarily through the renal pathway. LNCaP tumor xenografts were clearly visualized by [^68^Ga]Ga-AV01084 and [^68^Ga]Ga-AV01088 but not visualized by [^68^Ga]Ga-AV02070 (Figure 3A). HEK293T:hFAP tumor xenografts were also visualized by the bispecific tracers ([^68^Ga]Ga-AV01084 and [^68^Ga]Ga-AV01088) but not visualized by [^68^Ga]Ga-PSMA-617 (Figure 3B). The bispecific tracers had bone and joint uptake, which is commonly seen for FAP-targeted tracers. A high kidney uptake could be observed in mice injected with the bispecific tracers and [^68^Ga]Ga-PSMA-617 but not in mice injected with [^68^Ga]Ga-AV02070. Co-injection of [^68^Ga]Ga-AV01088 with the PSMA inhibitor 2-PMPA (500 µg) reduced the uptake of [^68^Ga]Ga-AV01088 in LNCaP tumors to almost background level (Figure 3A), confirming the uptake is PSMA-mediated. Similarly, co-injection of [^68^Ga]Ga-AV01088 with FAPI-04 (250 µg) reduced the uptake of [^68^Ga]Ga-AV01088 in HEK293T:hFAP tumors to almost background level (Figure 3B), indicating that the uptake is FAP-mediated.

**Figure 4:**
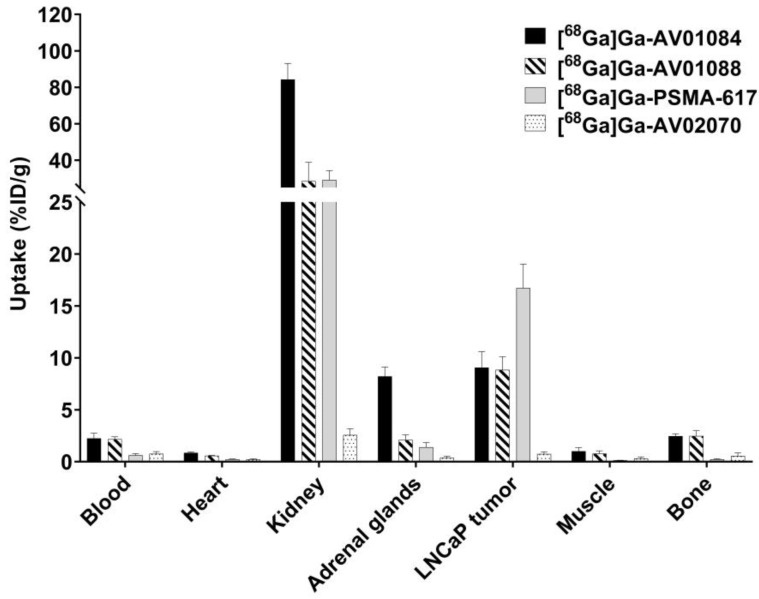
Biodistribution of [^68^Ga]Ga-AV01084, [^68^Ga]Ga-AV01088, [^68^Ga]Ga-PSMA-617, and [^68^Ga]Ga-AV02070 in LNCaP tumor-bearing mice. Error bars indicate standard deviation. The data of [^68^Ga]Ga-PSMA-617 have been reported previously [33] and are included here for comparison.

Biodistribution studies were conducted at 1 h post-injection with ^68^Ga-labeled AV01084, AV01088, and AV02070 in LNCaP tumor-bearing mice (Figure 4, Table S4), and the previously reported data from [^68^Ga]Ga-PSMA-617 obtained using the same tumor model are included for comparison [33]. The results were congruous with the observation from their PET images. The tumor uptake values of [^68^Ga]Ga-AV01084, [^68^Ga]Ga-AV01088, [^68^Ga]Ga-PSMA-617, and [^68^Ga]Ga-AV02070 were 9.05 ± 1.54, 8.85 ± 1.25, 16.7 ± 2.30, and 0.74 ± 0.21 %ID/g, respectively. The results showed very low tumor uptake in mice injected with [^68^Ga]Ga-AV02070, which indicates a very low FAP expression level in this tumor model. The uptake of these tracers in major organs and tissues has similar trends observed in their PET images of LNCaP tumor-bearing mice. The bispecific tracers ([^68^Ga]Ga-AV01084 and [^68^Ga]Ga-AV01088) have significantly higher blood and bone uptake values than the monospecific tracers, [^68^Ga]Ga-PSMA-617 and [^68^Ga]Ga-AV02070 (blood uptake: 1.56–2.26 vs. 0.28–0.75 %ID/g, *p* < 0.05; bone uptake: 0.88–2.47 vs. 0.09–0.56 %ID/g, *p* < 0.05). This results in a significantly lower tumor-to-blood ratio (4.09 ± 0.77 for [^68^Ga]Ga-AV01084, 4.10 ± 0.88 for [^68^Ga]Ga-AV01088 and 27.7 ± 6.28 for [^68^Ga]Ga-PSMA-617) and a lower tumor-to-bone ratio (3.70 ± 0.83 for [^68^Ga]Ga-AV01084, 3.75 ± 1.28 for [^68^Ga]Ga-AV01088, and 96.5 ± 47.6 for [^68^Ga]Ga-PSMA-617). [^68^Ga]Ga-AV01084 has higher heart, kidney, and adrenal gland uptake than [^68^Ga]Ga-AV01088 (Figure 4, *p* < 0.05).

**Figure 5:**
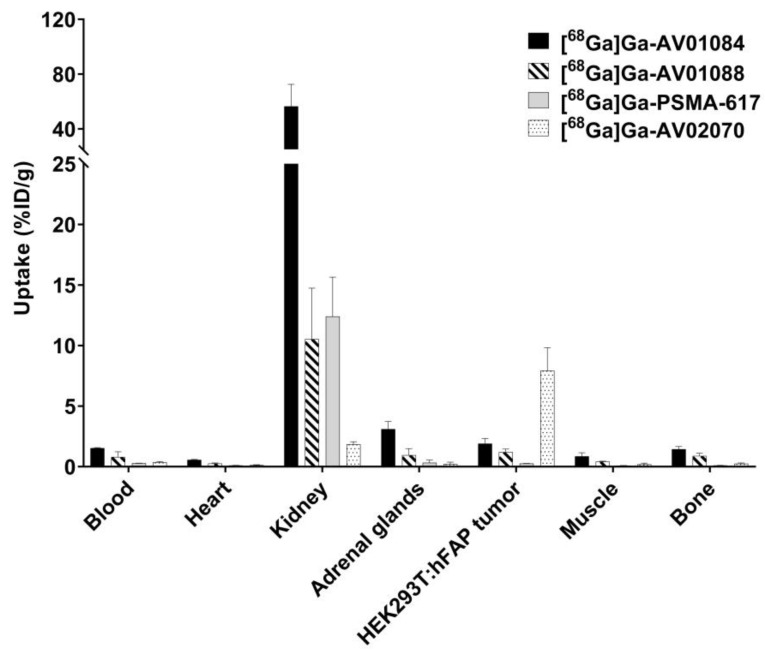
Biodistribution of [^68^Ga]Ga-AV01084, [^68^Ga]Ga-AV01088, [^68^Ga]Ga-PSMA-617 and [^68^Ga]Ga-AV02070 in HEK293T:hFAP tumor-bearing mice. Error bars indicate standard deviation. The data of [^68^Ga]Ga-AV02070 have been reported previously [31] and are included here for comparison.

Biodistribution studies were also conducted at 1 h post-injection with [^68^Ga]Ga-AV01084, [^68^Ga]Ga-AV01088, and [^68^Ga]Ga-PSMA-617 in HEK293T:hFAP tumor-bearing mice (Figure 5 and Table S5), and the previously reported data from [^68^Ga]Ga-AV02070 [31] using the same tumor model are included for comparison. Tumor uptake values of [^68^Ga]Ga-AV01084, [^68^Ga]Ga-AV01088, [^68^Ga]Ga-PSMA-617, and [^68^Ga]Ga-AV02070 were 1.90 ± 0.41, 1.20 ± 0.25, 0.26 ± 0.01, and 7.93 ± 1.88 %ID/g, respectively. The very low tumor uptake (0.26 ± 0.01 %ID/g) of [^68^Ga]Ga-PSMA-617 indicates that there is no significant PSMA expression in the HEK293T:hFAP tumor model. The uptake levels (Table S5) of these ^68^Ga-labeled tracers in major organs/tissues of HEK293T:hFAP tumor-bearing mice are congruous with the trends observed in their PET images (Figure 3B) acquired at 1 h post-injection.

**Figure 6:**
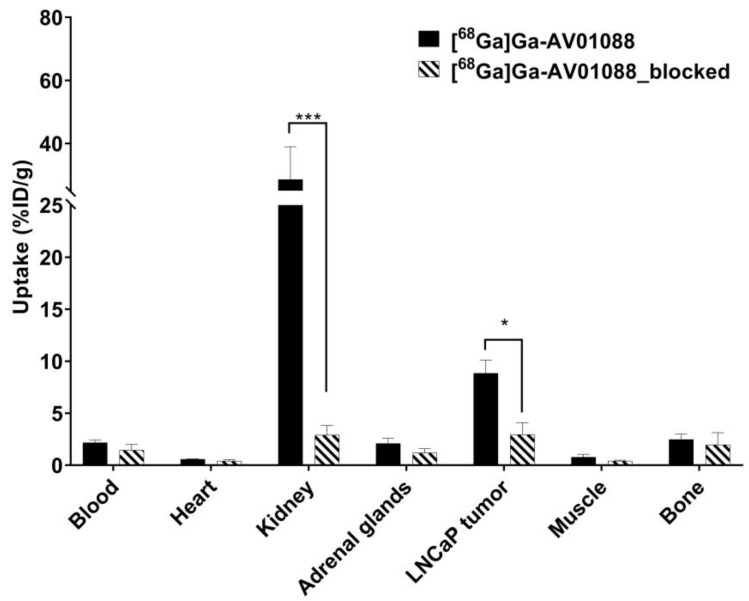
Comparison of [^68^Ga]Ga-AV01088 with and without co-injection of 2-PMPA on the uptake in LNCaP tumor xenografts and major organs/tissues in mice at 1 h post-injection. Error bars indicate standard deviation. * *p* < 0.05, *** *p* < 0.001.

Co-injection of 2-PMPA reduced the average uptake of [^68^Ga]Ga-AV01088 in LNCaP tumor xenografts by 67% (8.85 ± 1.25 %ID/g down to 2.95 ± 1.13 %ID/g at 1 h post-injection), confirming the specific uptake of [^68^Ga]Ga-AV01088 in LNCaP tumor xenografts (Figure 6). Co-injection of FAPI-04 reduced the average uptake of [^68^Ga]Ga-AV01088 in HEK293T:hFAP tumor xenografts by 57% (1.2 ± 0.25 %ID/g down to 0.51 ± 0.14 %ID/g at 1 h post-injection), demonstrating the specific uptake of [^68^Ga]Ga-AV01088 in HEK293T:hFAP tumor xenografts as well.

## 3. Discussion

Previously, we synthesized three ^68^Ga-labeled bispecific PSMA/FAP radiotracers, [^68^Ga]Ga-AV01017, [^68^Ga]Ga-AV01030, and [^68^Ga]Ga-AV01038 [28], which incorporate a quinoline-based FAP-targeted pharmacophore and an anthracene-based PSMA-targeted pharmacophore of [^68^Ga]Ga-HTK03041. The PSMA and FAP in vitro competition binding assays indicated that our bispecific tracers have lower binding affinity to PSMA compared to the monospecific PSMA-targeted ligand, Ga-HTK03041, but maintained a similar binding affinity to FAP when compared to the monospecific FAP-targeted ligand, Ga-FAPI-04. However, the bispecific tracers have higher uptake in major organs (blood, muscle, bone, and heart) and decreased tumor uptake in the mouse models in comparison to their ^68^Ga-labeled monospecific counterparts, [^68^Ga]Ga-HTK03041 and [^68^Ga]Ga-FAPI-04.

Therefore, in this report, we selected the pharmacophores of [^68^Ga]Ga-PSMA-617 (in brown, Figure 1) and [^68^Ga]Ga-AV02070 (in blue, Figure 1) for constructing our bispecific PSMA/FAP tracers as they are more hydrophilic and have a high affinity for PSMA and FAP, respectively. We separated the two pharmacophores with a Lys-Gly linker (Figure 1B). The PSMA-targeted pharmacophore (Lys(tranexamic acid-2-Nal)-urea-Glu and the linker (Lys-Gly) were constructed directly on solid phase using commercially available amino acids. For AV01084, the pyridine-based FAP-targeted ligand and the DOTA chelator were linked to the α-amino group and side-chain of Lys, respectively. While for AV01088, the DOTA chelator and the pyridine-based FAP-targeted ligand were coupled to the α-amino group and side-chain of Lys, respectively. This allowed us to investigate the effect of the position of the DOTA chelator on the binding affinity and biodistribution of the bispecific tracers.

The enzymatic assay (Figure 2B) showed that the FAP-binding affinities of Ga-AV01084 (IC_50_ = 10.9 ± 0.67 nM) and Ga-AV01088 (IC_50_ = 16.7 ± 1.53 nM) were comparable or even slightly better than that of the previously reported Ga-AV02070 (IC_50_ = 17.1 ± 4.60 nM) [31]. We also measured the FAP-binding affinity of Ga-PSMA-617 to determine whether the PSMA-targeted pharmacophore has any effect on the overall FAP binding of our bispecific ligands or not. The very weak binding affinity of Ga-PSMA-617 (IC_50_ > 1000 nM) indicates that the potent FAP-binding affinity of our bispecific ligands is mainly due to the binding of the AV02070 pharmacophore.

The PSMA-binding affinities of the bispecific ligands (K_i_ = 11.6–28.7 nM) were inferior to that of Ga-PSMA-617 (K_i_ = 1.23 ± 0.08) [33]. However, when compared to the bispecific tracers from our previous report (K_i_ = 20.1–54.4 nM), changing Ala(9-Anth) to 2-Nal and changing quinoline to pyridine significantly increased the binding affinity of bispecific PSMA/FAP tracers to PSMA. This supports our hypothesis that there might be an interaction between the pharmacophores that interferes with the overall binding of the bispecific ligands to PSMA. Moreover, Ga-AV02070 has a very minimal binding affinity to PSMA (K_i_ > 1000 nM), indicating that the PSMA-binding affinity of our bispecific ligands is mainly due to the binding of the PSMA-617 pharmacophore.

Despite having a lower uptake in the LNCaP tumor compared to [^68^Ga]Ga-PSMA-617 (16.7 ± 2.30 %ID/g), [^68^Ga]Ga-AV01084 and [^68^Ga]Ga-AV01088 (9.05 ± 1.54 and 8.85 ± 1.25 %ID/g, respectively) actually had better tumor uptake than our three previously reported bispecific PSMA/FAP tracers ([^68^Ga]Ga-AV01017, [^68^Ga]Ga-AV01030 and [^68^Ga]AV01038) (4.25–5.17 %ID/g) [28]. Moreover, co-injection of [^68^Ga]Ga-AV01088 with 2-PMPA decreased the tumor uptake by 67%, indicating that the tumor uptake is specific to PSMA. We also observed a significant bone uptake (1.97 ± 1.15 %ID/g) when [^68^Ga]Ga-AV01088 was co-injected with 2-PMPA, whereas there was a decreased bone uptake to the background level (0.20 ± 0.06 %ID/g) when [^68^Ga]Ga-AV01088 was co-injected with FAPI-04. This indicates that the bone uptake of [^68^Ga]Ga-AV01088 might be due to the specific binding of the FAP-targeted pharmacophore. Uptake of [^68^Ga]Ga-AV01088 to HEK293T:hFAP tumor is also FAP-mediated, as demonstrated by the significantly decreased tumor uptake obtained when co-injected with FAPI-04.

Unlike the improved uptake in LNCaP tumor xenografts, the uptake of [^68^Ga]Ga-AV01084 and [^68^Ga]Ga-AV01088 (1.20–1.90 %ID/g) in HEK293T:hFAP tumor xenografts remains significantly lower than that of the monospecific counterpart, [^68^Ga]Ga-AV02070 (7.93 ± 1.88 %ID/g, *p* < 0.05). One possible reason is the addition of the succinic acid linker, which might have interfered with the binding of our tracers to FAP, thus decreasing the tumor uptake. Previous studies have shown the importance of piperazine-based linkers for maintaining the good tumor uptake of FAP-targeted tracers [34]. Therefore, incorporating a piperazine-based linker can also be considered for future modifications to improve the uptake of bispecific PSMA/FAP tracers to the FAP-expressing tumors.

Compared to the monospecific tracers, the bispecific tracers have higher blood, bone, and muscle uptake. However, the current modified tracers have significantly decreased blood retention (0.79–2.26 %ID/g) when compared to the bispecific PSMA/FAP tracers in our previous report (5.75–11.94 %ID/g) [28]. This leads to better LNCaP tumor-to-blood contrast ratios for [^68^Ga]Ga-AV01084 (4.09 ± 0.77) and [^68^Ga]Ga-AV01088 (4.10 ± 0.88) (Table S4) than our previously reported bispecific PSMA/FAP tracers (0.48–0.89) [28]. The decreased blood retention might be attributed to the increased hydrophilicity of the new bispecific tracers shown by their low LogD_7.4_ values (<−3.60). This is consistent with previous findings that more hydrophilic radiotracers will have faster pharmacokinetics and clearance and, hence, a better tumor-to-background contrast ratio [35]. In addition, there are no significant differences in the tumor uptake or the tumor-to-background contrast ratios between [^68^Ga]Ga-AV01084 and [^68^Ga]Ga-AV01088, which indicates that the position of the DOTA chelator at the lysine linker does not have a crucial effect on the pharmacokinetics of the tracers.

**Figure 7:**
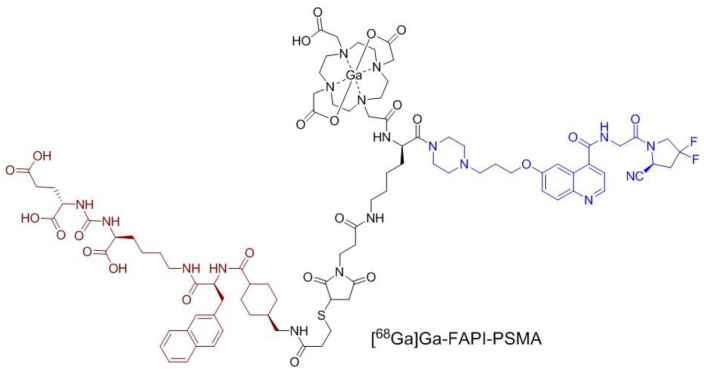
The chemical structure of [^68^Ga]Ga-FAPI-PSMA [27]. The PSMA- and FAP-targeted pharmacophores are shown in brown and blue, respectively.

Previously, Wang et al. [27] reported a ^68^Ga-labeled bispecific PSMA/FAP tracer, [^68^Ga]Ga-FAPI-PSMA (Figure 7), consisting of a PSMA-targeted pharmacophore from PSMA-617 (in brown, Figure 7) and an FAP-targeted pharmacophore from FAPI-04 (in blue). It would be difficult to directly compare the performance of our bispecific radiotracers with [^68^Ga]Ga-FAPI-PSMA as different tumor models were used for evaluation: PSMA-expressing LNCaP tumors and FAP-expressing HEK293T:hFAP tumors were used in our study, and PSMA-expressing 22Rv1 tumors and FAP-expressing U87 MG tumors were used by Wang et al. [27]. [^68^Ga]Ga-FAPI-PSMA was shown to achieve superior tumor uptake (SUVmax = 1.67 for U87 MG tumor xenografts; SUVmax = 1.32 for 22Rv1 tumor xenografts) when compared to those of the monospecific tracers, [^68^Ga]Ga-FAPI-04 (SUVmax = 0.45 for U87 MG tumor xenografts) and [^68^Ga]Ga-PSMA-617 (SUVmax = 0.25 for 22Rv1 tumor xenografts). This demonstrates the potential of utilizing bispecific PSMA/FAP tracers to improve the tumor uptake. However, similar to our results, [^68^Ga]Ga-FAPI-PSMA also had higher background uptake compared to the monospecific tracers.

Replacing the quinoline-based FAP-targeted pharmacophore with a pyridine-based FAP-targeted pharmacophore helps decrease the background uptake (blood, bone, and muscle) of the bispecific tracers; however, it also results in a significantly lower uptake in HEK293T:hFAP tumors. Therefore, future attempts on the use of a pyridine-based FAP-targeted pharmacophore for the design of bispecific PSMA/FAP tracers need to include the optimization of linkers such as incorporating a piperazine-based linker to continue improving FAP-binding affinity and tumor uptake.

## 4. Materials and Methods

### 4.1. Synthesis of Bispecific PSMA/FAP-Targeted Ligands

The procedures for the synthesis, purification, and characterizations of the bispecific PSMA/FAP ligands, AV01084 and AV01088, and their Ga-labeled analogs (both ^nat^Ga and ^68^Ga) are provided in the Supplementary Materials (Tables S1–S3 and Figures S1–S11).

### 4.2. Cell Culture

The HEK293T:hFAP cells were cultured in DMEM GlutaMAX™ medium (Thermo Fisher Scientific, Waltham, MA, USA) supplemented with 10% FBS, penicillin (100 U/mL), and streptomycin (100 μg/mL) at 37 °C in the presence of 5% CO_2_ [28]. The prostate cancer LNCaP cells obtained from ATCC (via Cedarlane, Burlington, ON, Canada) were cultured in RPMI 1640 medium supplemented with 10% FBS, penicillin (100 U/mL), and streptomycin (100 μg/mL). After reaching 80–90% confluence, the cells were washed with pH 7.4 phosphate-buffered saline (PBS) and collected after trypsinization (1 min).

### 4.3. Lipophilicity Measurement

The lipophilicity of ^68^Ga-labeled tracers was determined by measuring their LogD_7.4_ values following literature procedures [31,36]. Briefly, ^68^Ga-labeled tracer (50 µL) was mixed with 3 mL *n*-octanol and 3 mL PBS (pH 7.4) in a 15 mL falcon tube. After vortexing (1 min) and centrifugation (3000 rpm, 15 min), 1 mL from each phase was collected and counted using a Perkin Elmer (Waltham, MA, USA) Wizard2 2480 gamma counter. The LogD_7.4_ value was calculated using the following equation: LogD_7.4_ = log_10_ [(counts in the *n*-octanol phase)/(counts in the buffer phase)].

### 4.4. In Vitro PSMA Competition Binding Assay

Binding affinities of Ga-labeled ligands to PSMA were measured following literature procedures using PSMA-expressing prostate cancer LNCaP cells [28,33]. K_i_ values were calculated using the nonlinear regression algorithm of GraphPad Prism 7.02 (San Diego, CA, USA) software.

### 4.5. In Vitro FAP Fluorescence Assay

Binding affinities of Ga-labeled ligands to FAP were measured following literature procedures [28,31]. Recombinant human FAP (0.2 µg/mL, 50 µL; BioLegend, San Diego, CA, USA) was added to a costar 96-well plate. Varied concentrations (0.2 pM to 2 µM) of Ga-labeled ligands and PBS were added to each well (in duplicate). After a 30 min incubation at 37 °C, Suc-Gly-Pro-AMC (50 µL, 2 µM; Bachem, Bubendorf, Switzerland) was added to each well. The fluorescent signals were acquired at pre-determined time points (15, 30, 45, and 60 min) using a FlexStation 3 Multi-Mode Microplate Reader (Molecular Devices, San Jose, CA, USA). The IC_50_ values were calculated using the “nonlinear fit model” of the GraphPad Prism 7.02 software.

### 4.6. Biodistribution and PET/CT Imaging Studies

Imaging and ex vivo biodistribution studies were performed following literature procedures [28,31,33] using male NRG (NOD.Cg-Rag1^tm1Mom^ Il2rg^tm1Wjl^/SzJ) mice. These animal experiments were performed following the guidelines of the Canadian Council on Animal Care and approved by the Animal Ethics Committee of the University of British Columbia. Mice were sedated by isoflurane (2.5% in oxygen) and injected with 100 µL LNCaP (2 × 10^5^ cells/mouse) or HEK293T:hFAP (8.5 × 10^6^ cells/mouse) cells subcutaneously behind the left shoulder. When tumors reached ~5–8 mm in diameter (~3–4 weeks for HEK293T:hFAP tumors and ~4–5 weeks for LNCaP tumors), mice were used for PET/CT imaging and ex vivo biodistribution studies.

For PET imaging studies, each mouse was injected with the ^68^Ga-labeled tracer (~4–6 MBq/mouse) through a tail vein while under anesthesia (2.5% isoflurane in oxygen). PET/CT imaging was conducted using a Siemens (Knoxville, TN, USA) Inveon micro PET/CT scanner. At 50 min post-injection, a 10 min CT scan was carried out for localization and attenuation correction, followed by a 10 min static PET imaging acquisition.

For biodistribution studies, the tumor-bearing mice were injected with ~2–4 MBq/mouse of the ^68^Ga-labeled tracer. For blocking studies, the mice bearing LNCAP tumor xenografts were co-injected with 2-PMPA (500 μg/mouse), and the mice bearing HEK293T:hFAP tumor xenografts were co-injected with FAPI-04 (250 μg/mouse). The tumor-bearing mice were euthanized by CO_2_ inhalation at 1 h post-injection, and organs/tissues of interest were collected and counted using a Perkin Elmer (Waltham, MA, USA) Wizard2 2480 gamma counter.

### 4.7. Statistical Analysis

Data analyses were conducted using the GraphPad Prism version 7.02 and Microsoft (Redmond, WA, USA) Excel 2016 software. All organs in the biodistribution studies of [^68^Ga]Ga-AV01084 and [^68^Ga]Ga-AV01088 in LNCaP and HEK293T:hFAP tumor-bearing mice were analyzed with one-way ANOVA and multiple *t*-tests. When the adjusted *p*-value was <0.05 using the Holm–Sidak method, a statistically significant difference was considered present.

## 5. Conclusions

Both bispecific tracers, [^68^Ga]Ga-AV01084 and [^68^Ga]Ga-AV01088, were confirmed to have the ability to bind PSMA and FAP in vitro and in vivo. Both [^68^Ga]Ga-AV01084 and [^68^Ga]Ga-AV01088 have decreased PSMA-binding affinities but retain comparable binding affinities towards FAP when compared with the monospecific tracers. Compared with three of our previously reported bispecific PSMA/FAP tracers ([^68^Ga]Ga-AV01017, [^68^Ga]Ga-AV01030, and [^68^Ga]Ga-AV01038), both [^68^Ga]Ga-AV01084 and Ga-AV01088 have better PSMA-binding affinity and improved tumor uptake in PSMA-expressing xenografts and tumor-to-background (blood, muscle, and bone) contrast ratios. Further optimization on the selection of linkers should be explored to improve the binding affinity, pharmacokinetics, and tumor uptake of the bispecific PSMA/FAP tracers.

## Data Availability

The data presented in this study are available in the Supplementary Materials.

## Supplementary Materials

The following supporting information can be downloaded at https://www.mdpi.com/article/10.3390/molecules29040800/s1. Detailed synthetic procedures and results for the preparation of bispecific PSMA/FAP ligands and their ^nat^Ga/^68^Ga-complexed analogs [29,31,33,36,37,38]; Table S1: HPLC purification conditions and MS characterizations of radiolabeling precursors; Table S2: HPLC purification conditions and MS characterizations of nonradioactive Ga-complexed standards of PSMA/FAP-targeted bispecific ligands; Table S3: HPLC conditions for the purification and quality control of ^68^Ga-labeled PSMA/FAP-targeted bispecific tracers; Table S4: Biodistribution and uptake ratios of ^68^Ga-labeled bispecific PSMA/FAP tracers, PSMA-617 and AV02070 in LNCaP tumor-bearing mice; Table S5: Biodistribution and uptake ratios of ^68^Ga-labeled bispecific PSMA/FAP tracers, PSMA-617, and AV02070 in HEK293T:hFAP tumor-bearing mice; Figure S1: MS spectrum of *tert*-butyl (2,3,5,6-tetrafluorophenyl) succinate (**2**); Figure S2: ^1^H NMR spectrum of *tert*-butyl (2,3,5,6-tetrafluorophenyl) succinate (**2**); Figure S3: MS spectrum of *tert*-butyl (*S*)-4-((2-((4-((2-(2-cyano-4,4-difluoropyrrolidin-1-yl)-2-oxoethyl)carbamoyl)pyridin-2-yl)(methyl)amino)ethyl)(methyl)amino)-4-oxobutanoate (**4**); Figure S4: ^1^H NMR spectrum of *tert*-butyl (*S*)-4-((2-((4-((2-(2-cyano-4,4-difluoropyrrolidin-1-yl)-2-oxoethyl)carbamoyl)pyridin-2-yl)(methyl)amino)ethyl)(methyl)amino)-4-oxobutanoate (**4**); Figure S5: MS spectrum of (*S*)-4-((2-((4-((2-(2-cyano-4,4-difluoropyrrolidin-1-yl)-2-oxoethyl)carbamoyl)pyridin-2-yl)(methyl)amino)ethyl)(methyl)amino)-4-oxobutanoic acid (**5**); Figure S6: ^1^H NMR spectrum of (*S*)-4-((2-((4-((2-(2-cyano-4,4-difluoropyrrolidin-1-yl)-2-oxoethyl)carbamoyl)yridine-2-yl)(methyl)amino)ethyl)(methyl)amino)-4-oxobutanoic acid (**5**); Figure S7: MS spectrum of AV01084; Figure S8: MS spectrum of AV01088; Figure S9: MS spectrum of Ga-AV01084; Figure S10: MS spectrum of Ga-AV01088; Figure S11: Representative QC radio-HPLC chromatograms of [^68^Ga]Ga-AV02084 and [^68^Ga]Ga-AV02088.
