# Supplementary material for: Synthesis and Preclinical Evaluation of Two Novel 68Ga-Labeled Bispecific PSMA/FAP-Targeted Tracers with 2-Nal-Containing PSMA-Targeted Pharmacophore and Pyridine-Based FAP-Targeted Pharmacophore
Source: Molecules. 2024 Feb 9;29(4):800. doi: 10.3390/molecules29040800 (PMC10892057; doi:10.3390/molecules29040800)
Supplement: Supplementary file 1 [file molecules-29-00800-s001.zip › molecules-2854182-supplementary.pdf]

SUPPLEMENTARY MATERIALS

General Methods

1-(1,1-Dimethylethyl) butanedioate (**1**), (*S*)-*N*-(2-(2-cyano-4,4-difluoropyrrolidin-1-yl)-2-oxoethyl)-2-[[2-[[(*tert*-butoxy)carbonyl]methylamino]ethyl]methylamino]pyridine-4-carboxamide (**3**), AV02070, Ga-AV02070, [^{68}Ga]Ga-AV02070, PSMA-617, Ga-PSMA-617, and [^{68}Ga]Ga-PSMA-617 were synthesized following literature procedure [1–4]. All other chemicals and solvents were obtained from commercial sources and used without further purification. The purification and quality control of DOTA-conjugated ligands and their ^{nat}Ga/^{68}Ga-complexed analogs were performed on Agilent (Santa Clara, CA, USA) HPLC systems equipped with a model 1200 quaternary pump, a model 1200 UV absorbance detector (220 nm), and a Bioscan (Washington, DC, USA) NaI scintillation detector. The HPLC columns used were a semi-preparative column (Luna C18, 5 μm, 250 × 10 mm) and an analytical column (Luna C18, 5 μm, 250 × 4.6 mm) purchased from Phenomenex (Torrance, CA, USA). The collected HPLC eluates containing the desired products were lyophilized using a Labconco (Kansas City, MO, USA) FreeZone 4.5 Plus freeze-drier. ^{1}H NMR acquisitions were taken by a Bruker (Billerica, MA, USA) Avance 300 MHz NMR spectrometer. MS analyses were conducted using the Waters (Milford, MA, USA) Acquity QDa mass spectrometer with the equipped 2489 UV/Vis detector and e2695 Separations module. C18 Sep-Pak cartridges (1 cm^{3}, 50 mg) were purchased from Waters (Milford, MA, USA). ^{68}Ga was eluted from an ITM Medical Isotopes GmbH (Munich, Germany) generator, and purified according to the previously published procedures using a DGA resin column from Eichrom Technologies LLC (Lisle, IL, USA) [5]. The radioactivity of ^{68}Ga-labeled tracers was measured using a Capintec (Ramsey, NJ, USA) CRC®-25R/W dose calibrator and the radioactivity of mouse tissues collected from biodistribution studies was counted using a Perkin Elmer (Waltham, MA, USA) Wizard2 2480 automatic gamma counter.

Synthesis of the FAP-targeted Ligand

Synthesis of *tert*-butyl (2,3,5,6-tetrafluorophenyl) succinate (**2**)

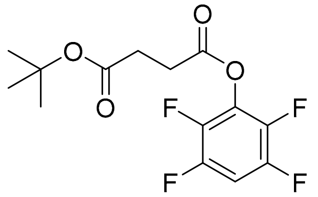

A solution of 1-(1,1-dimethylethyl) butanedioate (**1**, 1.87 g, 10.7 mmol) in dichloromethane (DCM) was added 2,3,5,6-tetrafluorophenol (TFP, 2.32 g, 13.9 mmol) and *N*-ethyl-*N'*-(3-dimethylaminopropyl)carbodiimide hydrochloride (EDC.HCl, 2.26 g, 11.8 mmol) and stirred at room temperature for 20 h. The solution was evaporated under reduced pressure, and the residue was dissolved in ethyl acetate (100 mL), washed with H_2O (100 mL × 2). The organic phase was collected, dried over anhydrous $MgSO_4$ and evaporated under reduced pressure to yield **2** (7.5 mmol, 71% yield) as light yellow oil, which was used for the next step without further purification. MS (ESI) calculated for $C_{14}H_{14}F_4O_4$ 322.1, found $[M+Na]^+$ 345.1. ^{1}H NMR (300

MHz, DMSO-d$_6$) δ 7.94 (tt, *J* = 11.0, 7.4 Hz, 1H), 3.02 – 2.92 (m, 2H), 2.67 – 2.57 (m, 2H), 1.39 (s, 9H).

Synthesis of *tert*-butyl (*S*)-4-((2-((4-((2-(2-cyano-4,4-difluoropyrrolidin-1-yl)-2-oxoethyl)carbamoyl)pyridin-2-yl)(methyl)amino)ethyl)(methyl)amino)-4-oxobutanoate (**4**)

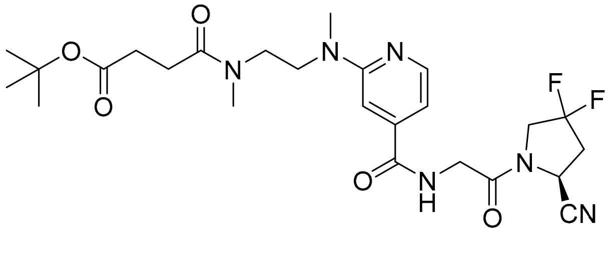

A solution of (*S*)-*N*-(2-(2-cyano-4,4-difluoropyrrolidin-1-yl)-2-oxoethyl)-2-[[2-[[(tert-butoxy)-carbonyl]methylamino]ethyl]methylamino]pyridine-4-carboxamide (**3**) (1.49 g, 3.1 mmol) in DCM (20 mL) was added TFA (20 mL) and stirred at room temperature for 2 h. After evaporation, the residue was dissolved in CH_3CN (50 mL), and compound **2** (2.44 g, 7.5 mmol) and triethylamine (TEA, 2.8 mL, 20 mmol) were added. After stirring at 50 °C for 19 h, the volatile solvent was evaporated under reduced procedure, and the residue was purified with flash column chromatography eluted with ethyl acetate (0.6 L), followed by 19:1 (v/v) ethyl acetate/methanol (1.8 L) and 9:1 (v/v) ethyl acetate/methanol (1 L), consecutively. The product eluate fractions were combined and evaporated under reduced pressure to yield **4** as a yellow powder (750 mg, 45% yield). MS (ESI) calculated for $C_{25}H_{34}F_2N_6O_5$ 536.3, found $[M+H]^+$ 537.3. ^{1}H NMR (300 MHz, DMSO-d$_6$) δ 7.39 (d, *J* = 8.1 Hz, 1H), 7.15 (s, 1H), 7.06 (t, *J* = 6.0 Hz, 2H), 6.88 (t, *J* = 6.4 Hz, 1H), 5.07 (dd, *J* = 9.1, 3.0 Hz, 2H), 4.45 (dd, *J* = 9.6, 4.2 Hz, 1H), 4.26 – 3.88 (m, 5H), 3.77 (d, *J* = 6.1 Hz, 4H), 3.70 (dd, *J* = 17.3, 5.6 Hz, 1H), 2.90 (dt, *J* = 14.3, 9.1 Hz, 1H), 2.83 – 2.67 (m, 3H), 1.38 (s, 9H).

Synthesis of (*S*)-4-((2-((4-((2-(2-cyano-4,4-difluoropyrrolidin-1-yl)-2-oxoethyl)carbamoyl)pyridin-2-yl)(methyl)amino)ethyl)(methyl)amino)-4-oxobutanoic acid (**5**)

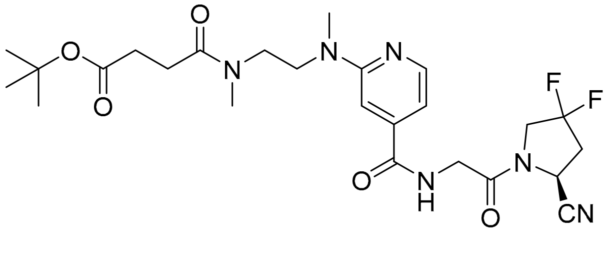

Compound **4** (737 mg, 1.4 mmol) was dissolved in DCM (10 mL), and triethylsilane (481 mg, 4.1 mmol) and TFA (10 mL) were added. After stirring for 2 h, the resulting solution was evaporated and the residue was dissolved in diethyl ether (250 mL). The diethyl ether solution was stirred for 2 h and the resulting yellow powder was filtered and dried under reduced pressure to yield 670 mg of **5**.xTFA. MS (ESI) calculated for $C_{21}H_{26}F_2N_6O_5$ 480.2, found $[M+H]^+$ 481.2. ^{1}H NMR (300 MHz, DMSO-d$_6$) δ 9.03 (dt, *J* = 27.2, 5.8 Hz, 1H), 8.27 – 8.12 (m, 3H), 7.25 (s,

1H), 7.11 – 6.97 (m, 2H), 5.10 (dd, *J* = 9.1, 2.9 Hz, 1H), 4.38 – 4.19 (m, 1H), 4.18 – 4.05 (m, 1H), 3.76 (s, 1H), 3.09 (s, 2H), 2.98 (s, 3H), 2.85 (s, 2H), 2.80 (s, 1H), 2.36 (dd, *J* = 8.7, 4.8 Hz, 3H).

Synthesis of DOTA-conjugated Peptides

AV01084 and AV01088 were synthesized on solid phase using Fmoc peptide chemistry. Fmoc-Lys(ivDde)-Wang resin (0.05 mmol, 0.081 g) was treated with 20% piperidine in DMF to remove the Fmoc protecting group. The isocyanate derivative of Glu(tBu)-OtBu (3 eq.) was synthesized following previously published procedures [6] and was added to the lysine-immobilized resin, with *N*,*N*-diisopropylethylamine (DIEA, 2 eq.) as the base and reacted for 16 h to form the Lys-urea-Glu moiety. The ivDde-protecting group was then removed with 2% hydrazine in DMF (5 × 5 min). Fmoc-2-Nal-OH (4 eq.), Fmoc-tranexamic acid (3 eq.), Fmoc-Gly-OH (4 eq.), and Fmoc-Lys(ivDde)-OH (4 eq.) were pre-activated with HATU (4 eq.) and DIEA (7 eq.) before being sequentially coupled to the resin. Following the removal of Fmoc protecting group, the FAP-targeted ligand (compound **5)** or DOTA-tris(*t*-butyl)ester (3 eq.) was pre-activated with HATU (3 eq.) and DIEA (7 eq.) and coupled to the α-amino group of lysine for AV01084 or AV01088, respectively. Finally, the ivDde-protecting group on lysine was removed and coupled with DOTA-tris(*t*-butyl)ester or compound **5** activated with HATU (3 eq.) and DIEA (7 eq.) for AV01084 and AV01088, respectively.

The peptides were deprotected and simultaneously cleaved from the resin with a mixture of trifluoroacetic acid (TFA, 95%), triisopropylsilane (TIS 2.5%) and water (2.5%) for 4 h at room temperature. The cleaved peptides were filtrated and then precipitated by the addition of cold diethyl ether. The crude peptides were collected by centrifugation and purified with HPLC (semi-preparative column; flow rate: 4.5 mL/min). The eluates containing the desired peptides were collected and lyophilized. The HPLC conditions, retention times, isolated yields and MS confirmations of DOTA-conjugated peptides are provided in Table S1.

Table S1: HPLC purification conditions and MS characterizations of radiolabeling precursors.

Compound name	HPLC conditions	Retention time (min)	Yield (%)	Calculated mass (m/z)	Found (m/z)
AV01084	26% CH_3CN and 0.1% TFA in H_2O	14.1	12	$[M+2H]^{2+}$ 845.4	$[M+2H]^{2+}$ 845.8
AV01088	26% CH_3CN and 0.1% TFA in H_2O	13.7	7.2	$[M+2H]^{2+}$ 845.4	$[M+2H]^{2+}$ 845.8

Synthesis of Nonradioactive Ga-complexed Standards

The nonradioactive Ga-complexed standards were prepared by reacting the DOTA-conjugated precursors with $GaCl_3$ (5 eq.) in NaOAc buffer (0.1 M, 500 µL, pH 4.2 – 4.5) at 80 °C for 15 min. The reaction mixture was then purified via HPLC (semi-preparative column, flow rate: 4.5 mL/min). The HPLC eluates containing the desired products were collected and lyophilized. The HPLC conditions, retention times, isolated yields and MS confirmations of these nonradioactive Ga-complexed standards are provided in Table S2.

Table S2: HPLC purification conditions and MS characterizations of nonradioactive Ga-complexed standards of PSMA/FAP-targeted bispecific ligands.

Compound name	HPLC conditions	Retention time (min)	Yield (%)	Calculated mass (m/z)	Found (m/z)
Ga-AV01084	27% CH_3CN and 0.1% TFA in H_2O	10.8	60	$[M+2H]^{2+}$ 878.9	$[M+2H]^{2+}$ 879.8
Ga-AV01088	26% CH_3CN and 0.1% TFA in H_2O	14.1	21	$[M+2H]^{2+}$ 878.9	$[M+2H]^{2+}$ 879.8

Synthesis of ^{68}Ga-labeled Tracers

The radiolabeling experiments were performed according to previously published procedures [3-4]. Purified $^{68}GaCl_3$ in 0.5 mL water was added to a 4-mL glass vial preloaded with 0.7 mL of HEPES buffer (2 M, pH 5.0) and 10 µL precursor solution (1 mM). The radiolabeling reaction was carried out under microwave heating for 1 min, followed by purification using the semi-preparative HPLC column. The eluate fraction containing the radiolabeled product was collected, diluted with water (50 mL), and passed through a C18 Sep-Pak cartridge that was pre-washed with ethanol (10 mL) and water (10 mL). The ^{68}Ga-labeled product was eluted off the cartridge with ethanol (0.4 mL), and diluted with PBS for imaging and biodistribution studies. Quality control was performed using the analytical column. The HPLC conditions and retention times are provided in Table S3. The tracers were obtained in 33 - 64% decay-corrected radiochemical yields with ≥ 44 GBq/µmol molar activity and > 95% radiochemical purity.

Table S3: HPLC conditions for the purification and quality control of ^{68}Ga-labeled PSMA/FAP-targeted bispecific tracers.

Tracer name	HPLC conditions		Retention time (min)
[^{68}Ga]Ga-AV01084	Semi-Prep	28% CH_3CN and 0.1% TFA in H_2O; flow rate 4.5 mL/min	16.8
	QC	31.5% CH_3CN and 0.1% TFA in H_2O; flow rate 2.0 mL/min	6.3
[^{68}Ga]Ga-AV01088	Semi-Prep	28% CH_3CN and 0.1% TFA in H_2O; flow rate 4.5 mL/min	16.7
	QC	31.5% CH_3CN and 0.1% TFA in H_2O; flow rate 2.0 mL/min	6.3

Table S4: Biodistribution (mean ± SD, n = 4) and uptake ratios of ^{68}Ga-labeled PSMA/FAP bispecific tracers, PSMA-617, and AV02070 in LNCaP tumor-bearing mice. The mice in the blocked group were co-injected with 2-PMPA (500 µg/mouse). The biodistribution data of [^{68}Ga]Ga-PSMA-617 have been reported previously by our group [2], and are included here for comparison.

Tissue (%ID/g)	[^{68}Ga]Ga-AV01084	[^{68}Ga]Ga-AV01088		[^{68}Ga]Ga-PSMA-617	[^{68}Ga]Ga-AV02070
	1 h	1 h	1 h blocked	1 h	1 h
Blood	2.26 ± 0.49	2.18 ± 0.22	1.45 ± 0.53	0.63 ± 0.13	0.75 ± 0.21
Fat	1.44 ± 0.15	0.70 ± 0.36	0.29 ± 0.20	0.25 ± 0.13	0.17 ± 0.16
Testes	0.66 ± 0.06	0.40 ± 0.13	0.34 ± 0.10	0.26 ± 0.06	0.38 ± 0.40
Small intestine	0.63 ± 0.15	0.52 ± 0.06	0.44 ± 0.21	0.31 ± 0.15	0.54 ± 0.17
Stomach	0.23 ± 0.06	0.20 ± 0.02	0.18 ± 0.01	0.08 ± 0.01	0.11 ± 0.05
Spleen	4.35 ± 1.25	1.14 ± 0.16	0.40 ± 0.09	1.18 ± 0.37	0.25 ± 0.07
Liver	0.49 ± 0.01	0.49 ± 0.08	0.40 ± 0.13	0.62 ± 0.15	0.47 ± 0.08
Pancreas	0.99 ± 0.07	0.70 ± 0.04	0.48 ± 0.20	0.19 ± 0.01	0.31 ± 0.21
Adrenal glands	8.22 ± 0.90	2.10 ± 0.48	1.19 ± 0.40	1.20 ± 0.57	0.36 ± 0.17
Kidney	84.3 ± 8.82	28.6 ± 10.4	2.91 ± 0.90	29.2 ± 5.13	2.57 ± 0.58
Lungs	2.09 ± 1.39	0.94 ± 0.64	1.03 ± 0.42	1.03 ± 0.32	0.63 ± 0.22
Heart	0.85 ± 0.08	0.57 ± 0.03	0.40 ± 0.14	0.23 ± 0.04	0.22 ± 0.05
LNCaP tumor	9.05 ± 1.54	8.85 ± 1.25	2.95 ± 1.13	16.7 ± 2.30	0.74 ± 0.21
Muscle	1.00 ± 0.35	0.77 ± 0.27	0.39 ± 0.08	0.12 ± 0.02	0.31 ± 0.14
Bone	2.47 ± 0.22	2.47 ± 0.50	1.97 ± 1.15	0.23 ± 0.05	0.56 ± 0.28
Brain	0.11 ± 0.05	0.06 ± 0.02	0.03 ± 0.01	0.02 ± 0.00	0.03 ± 0.01
Tumor/bone	3.70 ± 0.83	3.75 ± 1.28	1.78 ± 0.98	96.5 ± 47.6	1.63 ± 0.95
Tumor/muscle	9.57 ± 2.73	12.7 ± 5.32	7.25 ± 1.77	133 ± 19.5	2.76 ± 1.44
Tumor/blood	4.09 ± 0.77	4.10 ± 0.88	2.06 ± 0.34	27.7 ± 6.28	0.99 ± 0.12
Tumor/kidney	0.10 ± 0.02	0.34 ± 0.13	1.00 ± 0.12	0.63 ± 0.09	0.29 ± 0.08

Table S5: Biodistribution (mean ± SD, n = 4) and uptake ratios of ^{68}Ga-labeled PSMA/FAP bispecific tracers in HEK293T:hFAP tumor-bearing mice. The mice in the blocked group were co-injected with FAPI-04 (250 µg/mouse). The biodistribution data of [^{68}Ga]Ga-AV02070 have been reported previously by our group [3], and are included here for comparison.

Tissue (%ID/g)	[^{68}Ga]Ga-AV01084	[^{68}Ga]Ga-AV01088		[^{68}Ga]Ga-PSMA-617	[^{68}Ga]Ga-AV02070
	1 h	1 h	1 h blocked	1 h	1 h
Blood	1.53 ± 0.03	0.79 ± 0.43	0.68 ± 0.25	0.28 ± 0.02	0.36 ± 0.05
Fat	0.78 ± 0.16	0.20 ± 0.05	0.19 ± 0.09	0.09 ± 0.04	0.44 ± 0.81
Testes	0.54 ± 0.05	0.22 ± 0.05	0.33 ± 0.15	0.15 ± 0.03	0.17 ± 0.07
Small Intestine	0.45 ± 0.06	0.20 ± 0.02	0.26 ± 0.07	0.14 ± 0.02	0.42 ± 0.13
Stomach	0.11 ± 0.02	0.08 ± 0.01	0.08 ± 0.03	0.07 ± 0.08	0.08 ± 0.04
Spleen	3.89 ± 1.38	0.81 ± 0.15	0.63 ± 0.28	0.55 ± 0.13	0.25 ± 0.12
Liver	0.46 ± 0.17	0.17 ± 0.01	0.26 ± 0.09	0.11 ± 0.01	0.39 ± 0.05
Pancreas	0.71 ± 0.05	0.31 ± 0.04	0.21 ± 0.07	0.11 ± 0.03	0.34 ± 0.45
Adrenal glands	3.10 ± 0.62	0.96 ± 0.53	0.44 ± 0.06	0.32 ± 0.22	0.22 ± 0.15
Kidney	56.4 ± 16.1	10.5 ± 4.20	8.18 ± 3.98	12.4 ± 3.24	1.85 ± 0.21
Lungs	1.60 ± 0.10	0.55 ± 0.05	0.76 ± 0.26	0.34 ± 0.05	0.34 ± 0.06
Heart	0.56 ± 0.03	0.26 ± 0.04	0.18 ± 0.04	0.10 ± 0.00	0.12 ± 0.03
HEK293T:hFAP tumor	1.90 ± 0.41	1.20 ± 0.25	0.51 ± 0.14	0.26 ± 0.01	7.93 ± 1.88
Muscle	0.85 ± 0.30	0.41 ± 0.03	0.16 ± 0.12	0.08 ± 0.01	0.19 ± 0.10
Bone	1.44 ± 0.21	0.88 ± 0.22	0.20 ± 0.06	0.09 ± 0.02	0.23 ± 0.06
Brain	0.05 ± 0.00	0.40 ± 0.74	0.02 ± 0.01	0.01 ± 0.00	0.02 ± 0.02
Tumor/bone	1.02 ± 0.10	1.08 ± 0.31	2.58 ± 0.68	2.95 ± 0.55	34.3 ± 7.35
Tumor/muscle	1.88 ± 0.31	2.44 ± 1.08	4.07 ± 2.13	3.01 ± 0.66	45.7 ± 9.88
Tumor/blood	1.06 ± 0.21	2.26 ± 2.25	0.78 ± 0.09	0.94 ± 0.13	22.9 ± 10.1
Tumor/kidney	0.03 ± 0.02	0.15 ± 0.14	0.06 ± 0.01	0.02 ± 0.00	4.34 ± 1.36

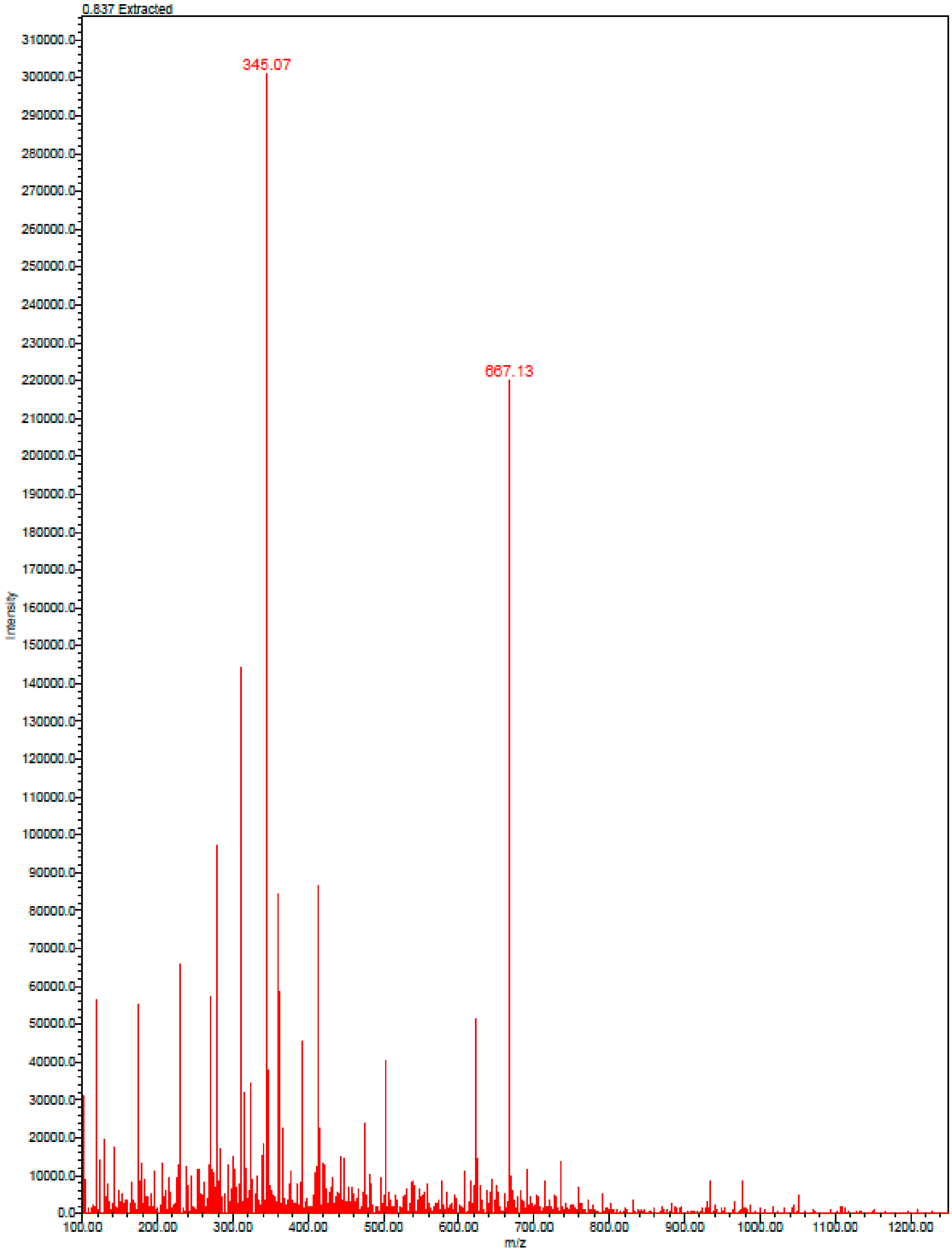

Figure S1: MS Spectrum of *tert*-butyl (2,3,5,6-tetrafluorophenyl) succinate (**2**).

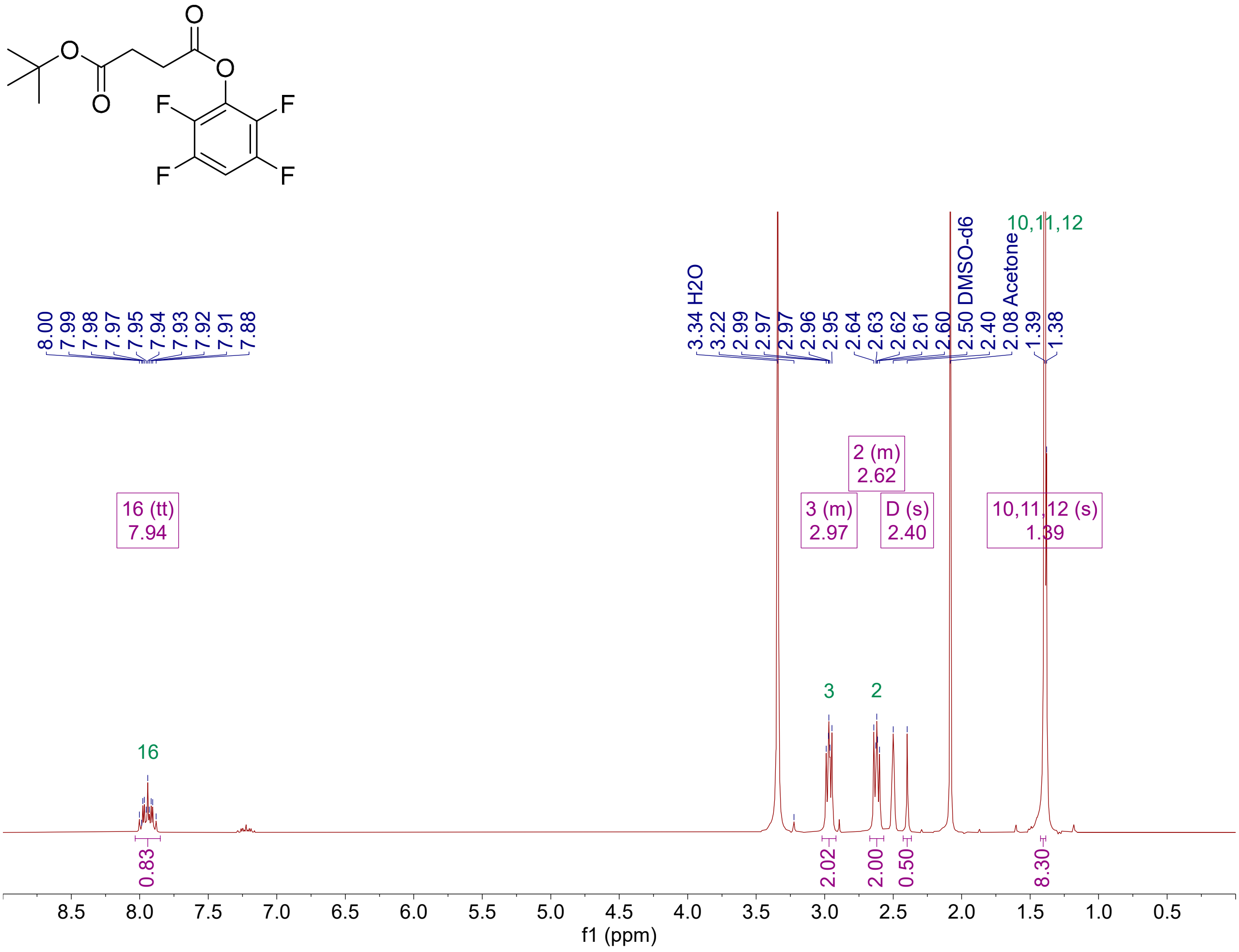

Figure S2: ^{1}H NMR Spectrum of *tert*-butyl (2,3,5,6-tetrafluorophenyl) succinate (**2**).

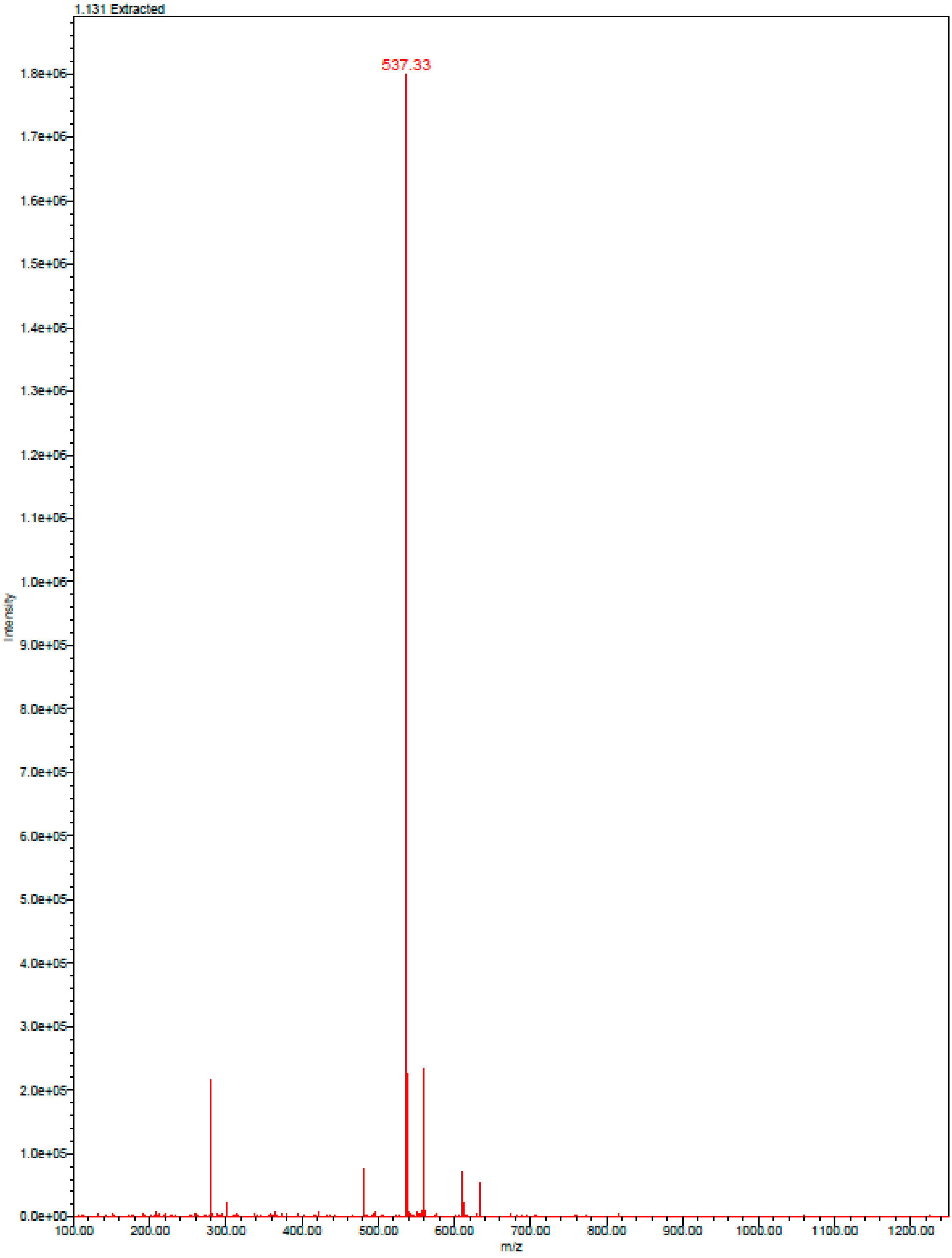

Figure S3: MS Spectrum of *tert*-butyl (*S*)-4-((2-((4-((2-(2-cyano-4,4-difluoropyrrolidin-1-yl)-2-oxoethyl)carbamoyl)pyridin-2-yl)(methyl)amino)ethyl)(methyl)amino)-4-oxobutanoate (**4**).

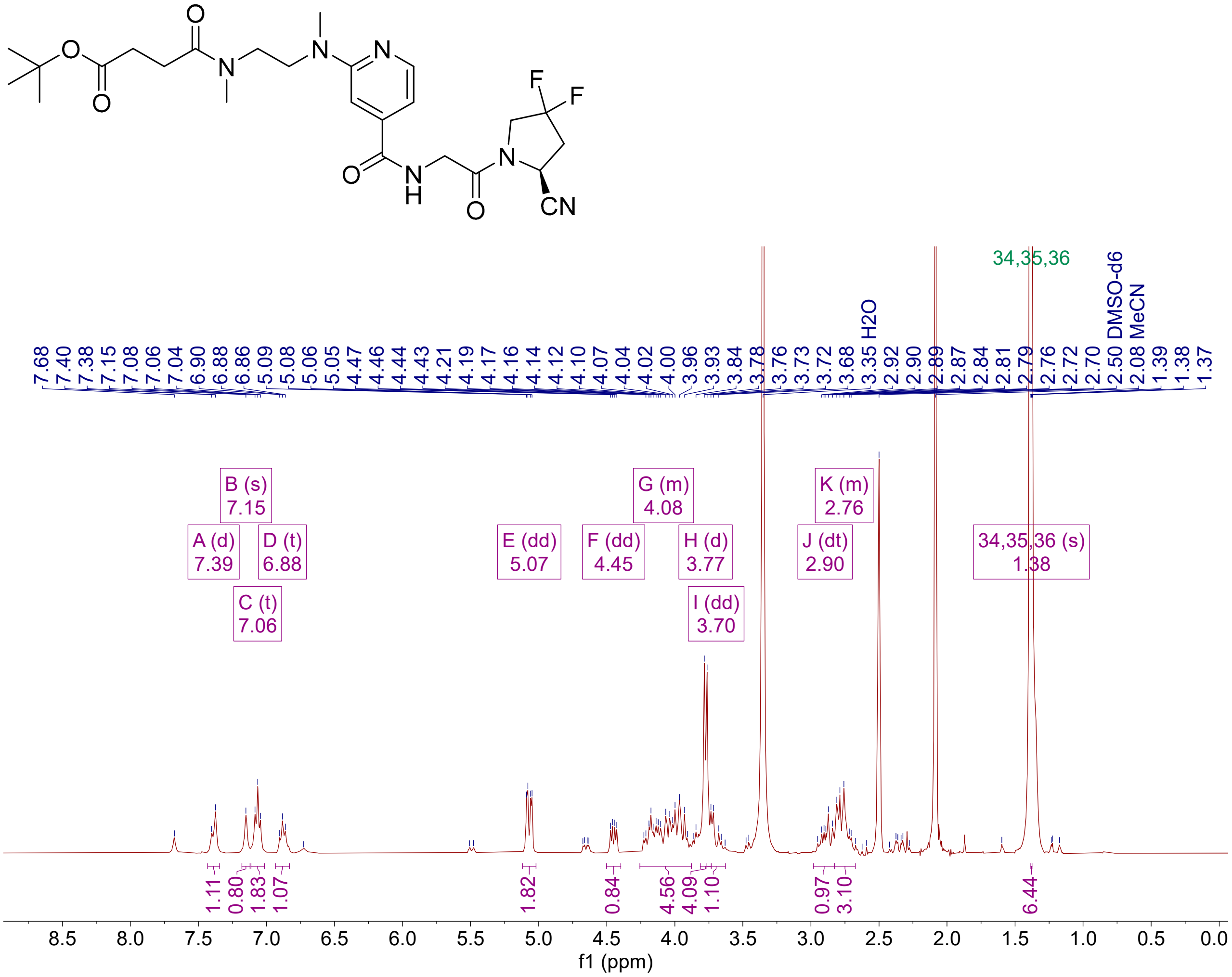

Figure S4: ^{1}H NMR Spectrum of *tert*-butyl (*S*)-4-((2-((4-((2-(2-cyano-4,4-difluoropyrrolidin-1-yl)-2-oxoethyl)carbamoyl)pyridin-2-yl)(methyl)amino)ethyl)(methyl)amino)-4-oxobutanoate (**4**).

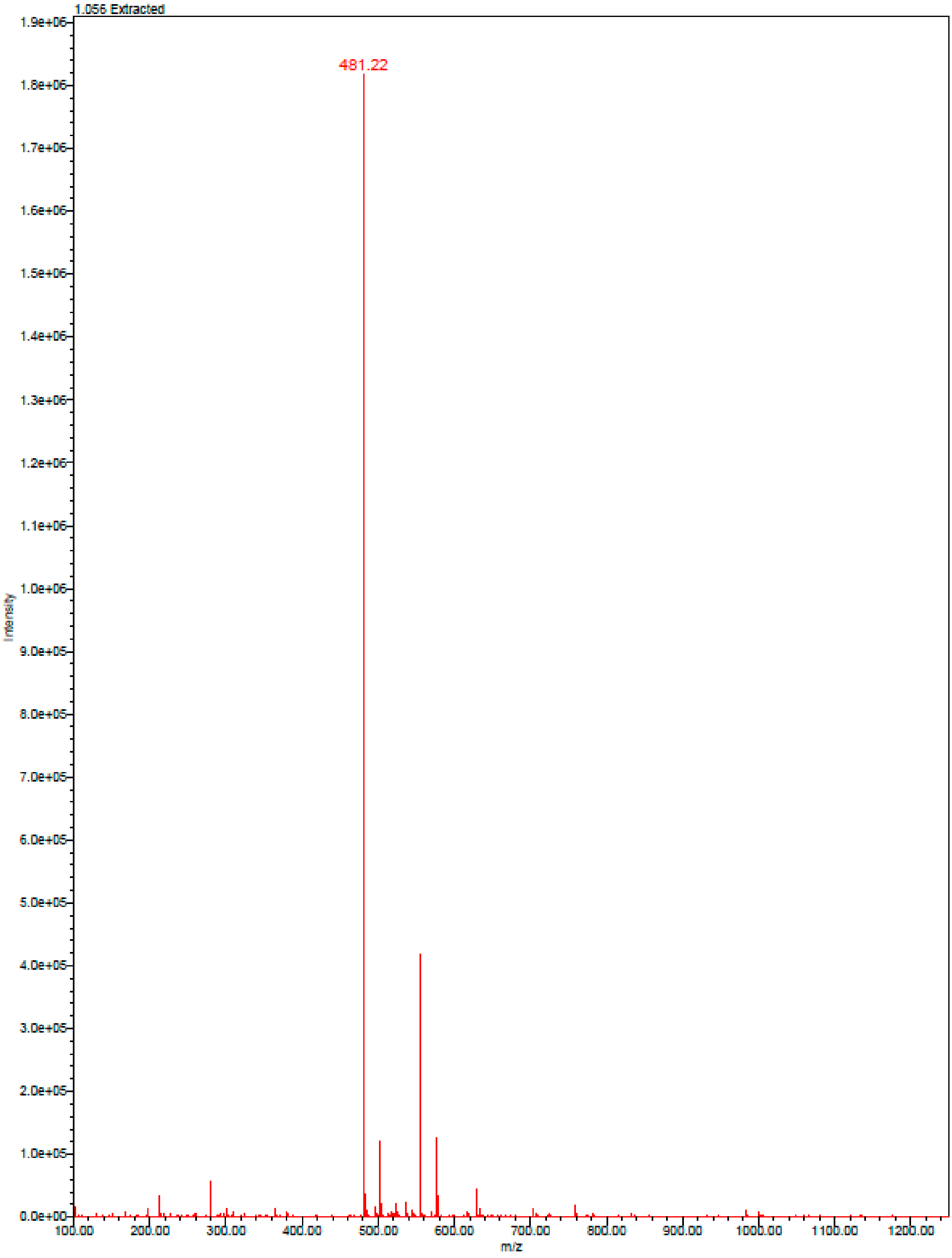

Figure S5: MS Spectrum of (*S*)-4-((2-((4-((2-(2-cyano-4,4-difluoropyrrolidin-1-yl)-2-oxoethyl)carbamoyl)pyridin-2-yl)(methyl)amino)ethyl)(methyl)amino)-4-oxobutanoic acid (**5**).

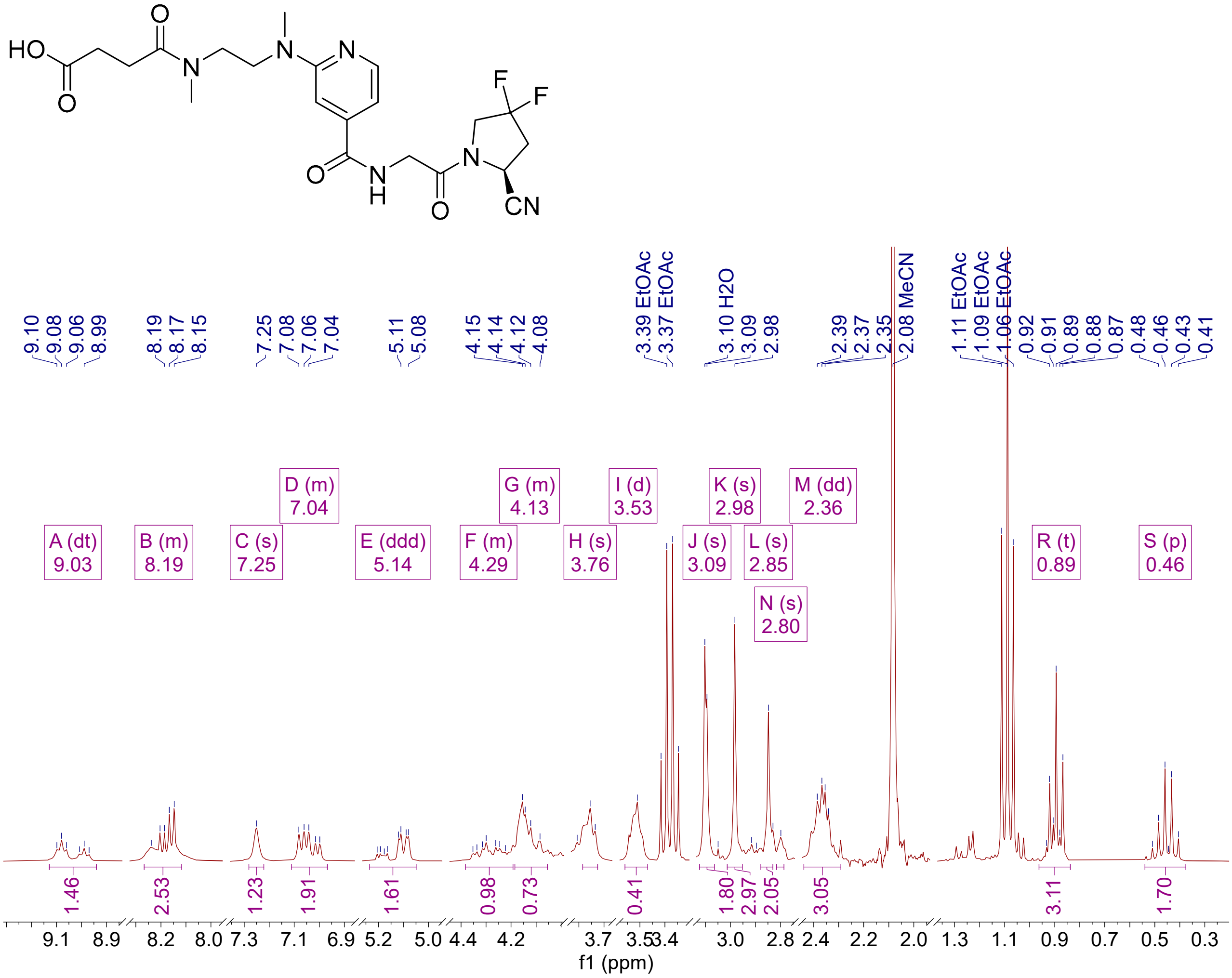

Figure S6: ^{1}H NMR Spectrum of (*S*)-4-((2-((4-((2-(2-cyano-4,4-difluoropyrrolidin-1-yl)-2-oxoethyl)carbamoyl)13yridine-2-yl)(methyl)amino)ethyl)(methyl)amino)-4-oxobutanoic acid (**5**).

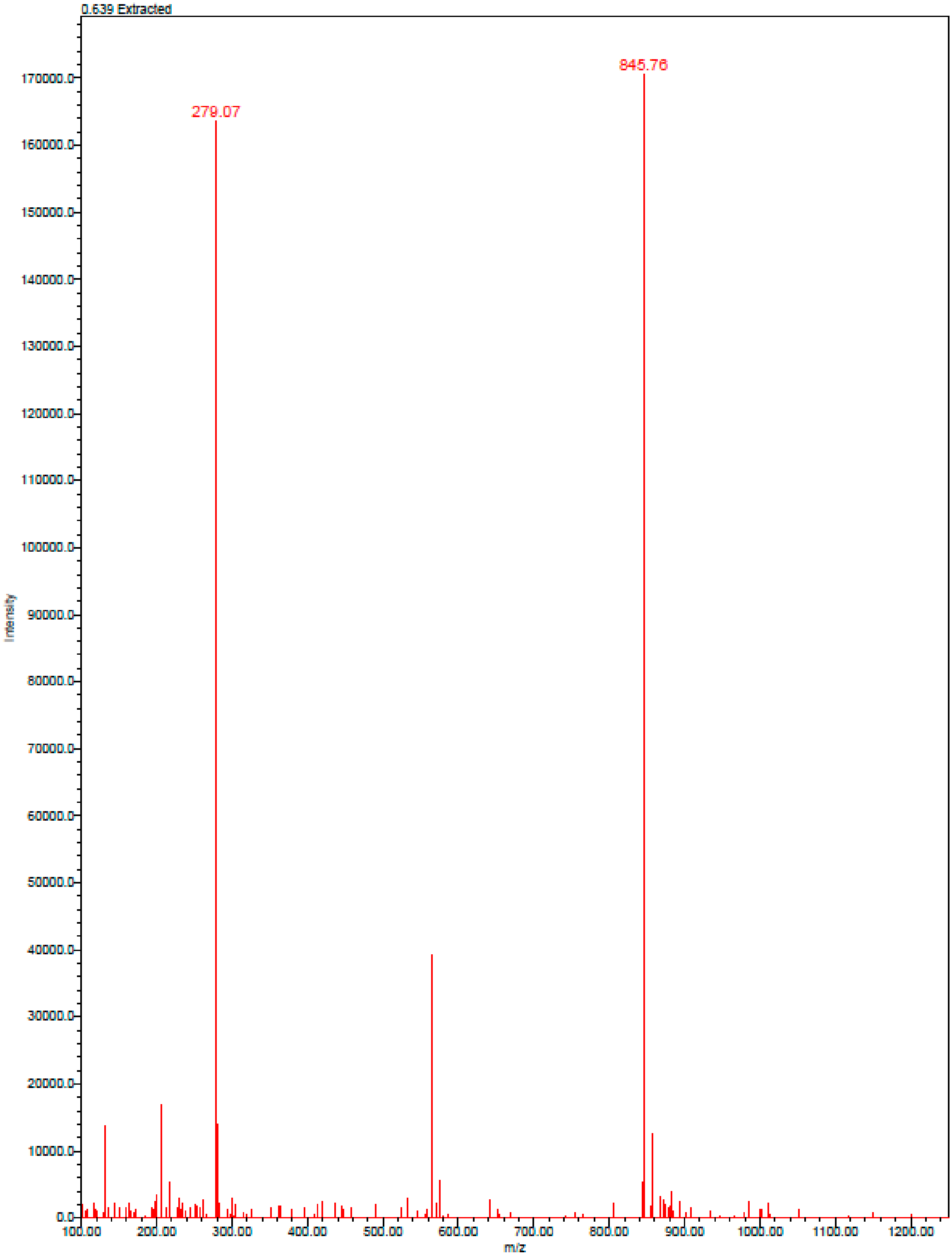

Figure S7: MS Spectrum of AV01084.

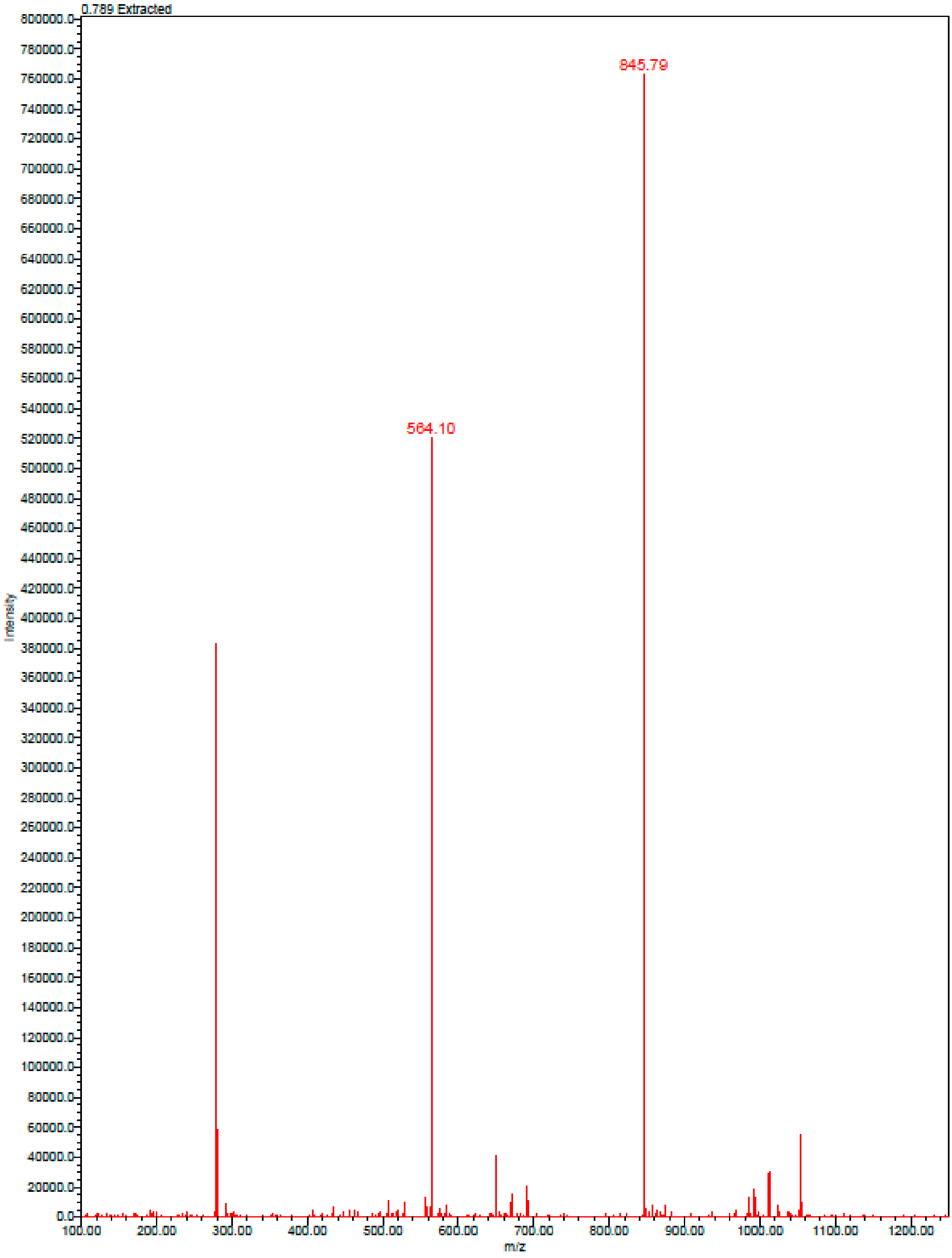

Figure S8: MS Spectrum of AV01088.

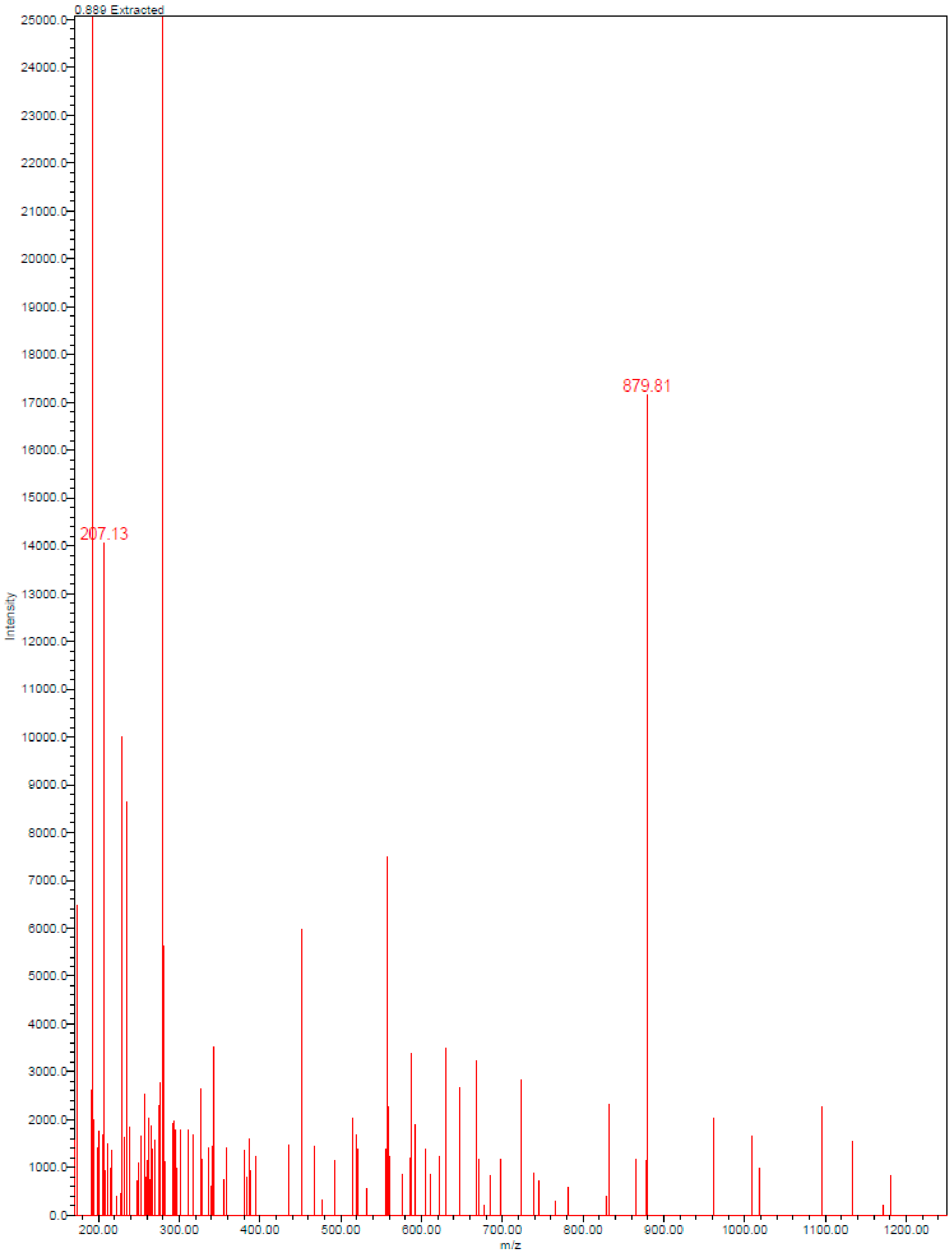

Figure S9: MS Spectrum of Ga-AV01084.

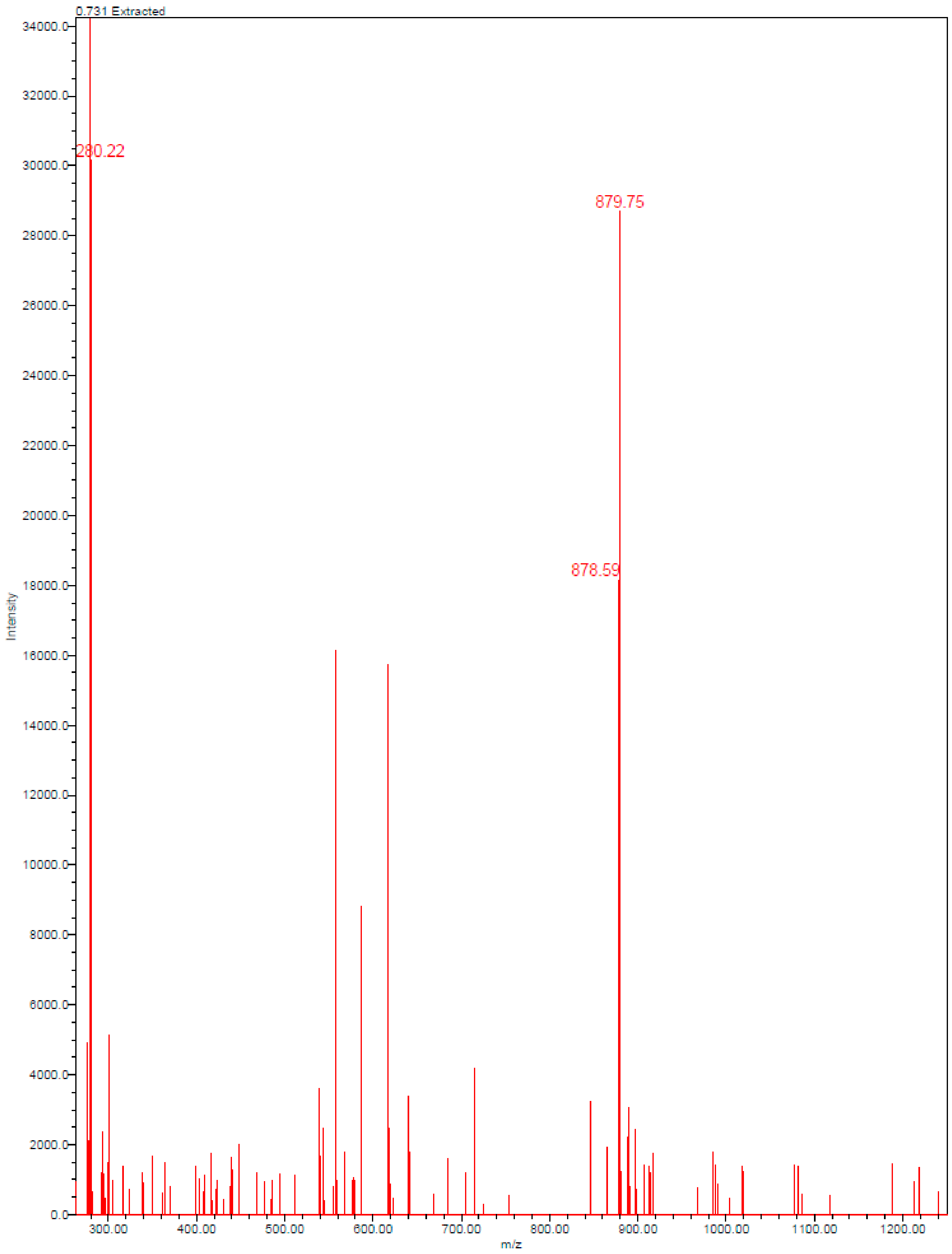

Figure S10: MS Spectrum of Ga-AV01088.

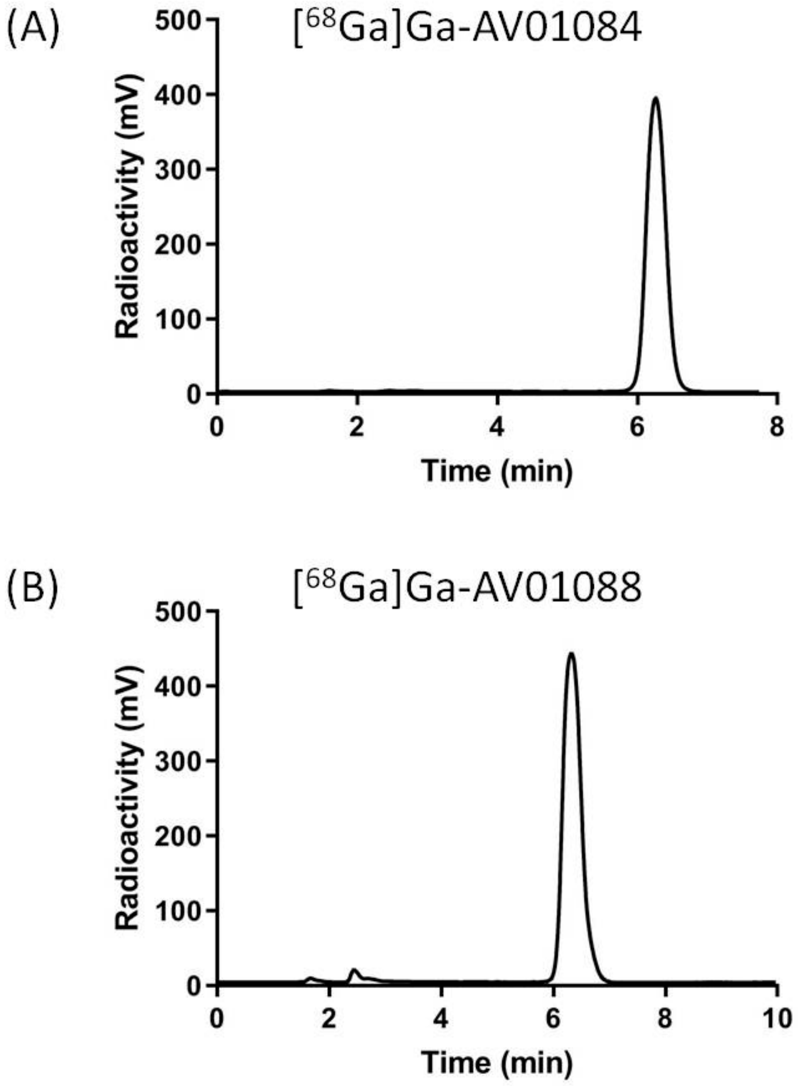

Figure S11: Representative QC radio-HPLC chromatograms of (A) [^{68}Ga]Ga-AV01084 and (B) [^{68}Ga]Ga-AV01088.

References

1. Jansen, K.; Heirbaut, L.; Verkerk, R.; Cheng, J. D.; Joossens, J.; Cos, P.; Maes, L.; Lambeir, A.-M.; De Meester, I.; Augustyns, K.; et al. Extended Structure–activity Relationship and Pharmacokinetic Investigation of (4-Quinolinoyl)glycyl-2-cyanopyrrolidine Inhibitors of Fibroblast Activation Protein (FAP). *J. Med. Chem.* **2014**, *57*, 3053–3074.
2. Kuo, H.-T.; Pan, J.; Zhang, Z.; Lau, J.; Merkens, H.; Zhang, C.; Colpo, N.; Lin, K.-S.; Bénard, F. Effects of Linker Modification on Tumor-to-kidney Contrast of ^{68}Ga-labeled PSMA-targeted Imaging Probes. *Mol. Pharmaceutics* **2018**, *15*, 3502–3511.
3. Verena, A.; Kuo, H.-T.; Merkens, H.; Zeisler, J.; Bendre, S.; Wong, A. A. W. L.; Bénard, F.; Lin, K.-S. Novel ^{68}Ga-labeled Pyridine-based Fibroblast Activation Protein-targeted Tracers with High Tumor-to-background Contrast. *Pharmaceuticals* **2023**, *16*, 449.
4. Wünsch, M.; Schröder, D.; Fröhr, T.; Teichmann, L.; Hedwig, S.; Janson, N.; Belu, C.; Simon, J.; Heidemeyer, S.; Holtkamp, P.; et al. Asymmetric Synthesis of Propargylamines as Amino Acid Surrogates in Peptidomimetics. *Beilstein J. Org. Chem.* **2017**, *13*, 2428–2441.
5. Lin, K.-S.; Pan, J.; Amouroux, G.; Turashvili, G.; Mesak, F.; Hundal-Jabal, N.; Pourghiasian, M.; Lau, J.; Jenni, S.; Aparicio, S.; et al. In Vivo Radioimaging of Bradykinin Receptor B1, a Widely Overexpressed Molecule in Human Cancer. *Cancer Res.* **2015**, *75*, 387–393.
6. Benešová, M.; Bauder-Wüst, U.; Schäfer, M.; Klika, K. D.; Mier, W.; Haberkorn, U.; Kopka, K.; Eder, M. Linker Modification Strategies to Control the Prostate-specific Membrane Antigen (PSMA)-targeting and Pharmacokinetic Properties of DOTA-conjugated PSMA Inhibitors. *J. Med. Chem.* **2016**, *59*, 1761–1775.
